# Morphological evolution of the mammalian jaw adductor complex

**DOI:** 10.1111/brv.12314

**Published:** 2016-11-23

**Authors:** Stephan Lautenschlager, Pamela Gill, Zhe‐Xi Luo, Michael J. Fagan, Emily J. Rayfield

**Affiliations:** ^1^ School of Earth Sciences University of Bristol Bristol BS8 1TQ U.K.; ^2^ School of Geography, Earth and Environmental Sciences University of Birmingham Birmingham B15 2TT U.K.; ^3^ Earth Science Department The Natural History Museum London SW7 5BD U.K.; ^4^ Department of Organismal Biology and Anatomy University of Chicago Chicago IL 60637 U.S.A.; ^5^ School of Engineering University of Hull Hull HU6 7RX U.K.

**Keywords:** mammalian evolution, Cynodontia, digital reconstruction, musculoskeletal evolution, vertebrate palaeontology, Mesozoic

## Abstract

The evolution of the mammalian jaw during the transition from non‐mammalian synapsids to crown mammals is a key event in vertebrate history and characterised by the gradual reduction of its individual bones into a single element and the concomitant transformation of the jaw joint and its incorporation into the middle ear complex. This osteological transformation is accompanied by a rearrangement and modification of the jaw adductor musculature, which is thought to have allowed the evolution of a more‐efficient masticatory system in comparison to the plesiomorphic synapsid condition. While osteological characters relating to this transition are well documented in the fossil record, the exact arrangement and modifications of the individual adductor muscles during the cynodont–mammaliaform transition have been debated for nearly a century.

We review the existing knowledge about the musculoskeletal evolution of the mammalian jaw adductor complex and evaluate previous hypotheses in the light of recently documented fossils that represent new specimens of existing species, which are of central importance to the mammalian origins debate. By employing computed tomography (CT) and digital reconstruction techniques to create three‐dimensional models of the jaw adductor musculature in a number of representative non‐mammalian cynodonts and mammaliaforms, we provide an updated perspective on mammalian jaw muscle evolution.

As an emerging consensus, current evidence suggests that the mammal‐like division of the jaw adductor musculature (into deep and superficial components of the m. masseter, the m. temporalis and the m. pterygoideus) was completed in Eucynodontia. The arrangement of the jaw adductor musculature in a mammalian fashion, with the m. pterygoideus group inserting on the dentary was completed in basal Mammaliaformes as suggested by the muscle reconstruction of *Morganucodon oehleri*. Consequently, transformation of the jaw adductor musculature from the ancestral (‘reptilian’) to the mammalian condition must have preceded the emergence of Mammalia and the full formation of the mammalian jaw joint. This suggests that the modification of the jaw adductor system played a pivotal role in the functional morphology and biomechanical stability of the jaw joint.

## I. INTRODUCTION

Modern mammals possess a unique suite of morphological and physiological characters which distinguishes them from other vertebrates. Distinct mammalian features include the presence of fur to maintain a constant body temperature, mammary glands to suckle their young and an enlarged brain and neocortex (Oftedal, 2002; Kemp, 2005). While there are several characteristics in the postcranial skeleton unique to mammals, such as differentiation of the vertebral series, the shoulder girdle and the limb muscles to permit parasagittal gait and agile locomotion (Bramble & Jenkins, 1989), many distinctive osteological features are focussed in the cranial skeleton. For example, (*i*) a mediolaterally expanded braincase to accommodate an enlarged brain, (*ii*) fusion of the palatal processes of the premaxilla and maxilla to form a secondary bony palate separating the oral and nasal cavities, (*iii*) a unique middle and inner ear morphology capable of high‐frequency sound detection, (*iv*) formation of a single bony housing (petrosal) for the inner ear, (*v*) an expanded, single bone (dentary) forming the lower jaw, (*vi*) a novel craniomandibular jaw joint formed by the squamosal and dentary, and (*vii*) a heterodont dentition differentiated into functional groups with a single (diphyodont) replacement phase between deciduous and permanent teeth (Kielan‐Jaworowska, Cifelli & Luo, 2004; Kemp, 2005; Rowe, Macrini & Luo, 2011; Manley, 2012). The sequence of hard‐tissue character transformations leading to the development of these characters is remarkably well documented in the fossil record, representing the evolution of a suite of correlated structural innovations rooted in modifications of the feeding and auditory systems (Allin & Hopson, 1992; Sidor & Hopson, 1998; Kemp, 2005; Luo, 2011). Throughout the evolutionary history of the group, from basal synapsids, to cynodonts and then early mammals, a step‐wise acquisition of these characters can be observed. The post‐dentary elements (articular, angular, surangular, prearticular, splenial) in the lower jaw were gradually reduced and partially lost, whereas some of the bones (articular, angular, quadrate) forming the ancestral (‘reptilian’) jaw joint were co‐opted into the ossicular chain (malleus, ectotympanic, incus). Concomitantly, a novel (‘mammalian’) jaw joint began to form between the expanded dentary and the squamosal *via* various transitional stages (Luo, 2011).

While a range of new fossil findings have substantiated our knowledge of such osteological transformations in recent years (e.g. Luo, Crompton & Sun, 2001; Luo, 2007; Zhou *et al.,* 2013), accompanying soft‐tissue modifications are considerably harder to track, but no less important. In particular, the jaw adductor musculature is thought to have undergone substantial changes in morphology and arrangement throughout the cynodont–mammal transition, which in turn made the evolution of a more‐efficient masticatory system, in comparison to the condition found in basal synapsids, possible. However, as muscle tissues have a very low preservation potential, many aspects of their anatomy can only be inferred from the preserved osteology. Consequently, it is unsurprising that although the functional aspects of the osteological and myological transition have been studied in detail for several decades, little is known about the exact arrangement of the individual adductor muscles. Accurate knowledge on the morphology of the jaw adductor system, however, is central to the debate on feeding and jaw joint–middle ear evolution across the cynodont–mammalian transition. Previously published muscle reconstructions have often been vague or conjectural and largely been restricted to two‐dimensional schematics (see also Section II). Here, we take advantage of recently documented fossils that represent new specimens of existing species, coupled with new species that are of central importance to the mammalian origins debate, to provide a revised perspective on the evolution of the mammalian jaw adductor system across the cynodont–mammaliaform transition. We employ computed tomography (CT) and digital restoration, reconstruction and modelling techniques to create three‐dimensional models of the hard tissue cranial anatomy and the resulting jaw adductor musculature in a number of representative non‐mammalian cynodonts and mammaliaforms. These detailed models are compared with previous hypotheses and used to decipher the sequence of musculoskeletal evolution leading from cynodonts to modern mammals.

## II. HISTORICAL ACCOUNT

The evolution of a novel jaw joint and mammalian middle ear arising from the ancestral quadrate–articular joint is a classic textbook example of an evolutionary transition and exaptation of anatomical structures. The associated modifications of hard‐ and soft‐tissue structures have intrigued palaeontologists, biologists and comparative anatomists for nearly two centuries. Reichert (1837), and later Gaupp (1913), were the first to recognise the homology of the reptilian jaw joint and the mammalian middle ear elements based on embryological studies. Facilitated by an already extensive collection of Permian and Triassic synapsids, Watson (1912) noted the arrangement of the adductor musculature in different taxa, including the Triassic cynodont *Cynognathus crateronotus*. He assumed a mammalian type of muscle insertion on the dentary and regarded the enlarged angle of the dentary as an indicator for the transfer of the jaw adductor muscles from the post‐dentary elements to the dentary, without however providing further detail on the exact attachment sites and muscle subdivisions. In a comprehensive study of the jaw adductor musculature across a wide range of extant and fossil vertebrates, Adams (1918) gave a more detailed account of the possible muscle origins and insertions of *Cynognathus*. His reconstruction included different subdivisions of a temporal muscle attaching to the enlarged coronoid process on the dentary, but also some muscles attaching on the articular and angular bones. For the following decades, Adams' (1918) reconstruction remained the most comprehensive until a number of new fossil findings revived the study of early mammals and their precursors. Newly discovered taxa and specimens, such as *Diarthrognathus* (Crompton, 1958, 1972), *Morganucodon* (Rigney, 1963), *Probainognathus* (1969a, 1970; Crompton, 1972) and others (Romer, 1967, 1969b; Crompton, 1972) provided detailed descriptions of transitional stages of jaw joint and inner and middle ear evolution. In addition, a better understanding of the osteology also sparked new interest in the reconstruction of jaw musculature in order to recognise functional modifications and evolutionary patterns across the cynodont–mammal transition. These reconstructions largely followed Adams' (1918) example of identifying muscle attachment sites based on comparisons with a range of extant vertebrates. In a series of publications, Barghusen (1968, 1972, 1973) reconstructed and illustrated jaw adductor muscle attachments for different basal synapsids and cynodonts. Based on these reconstructions, Barghusen (1968) inferred that the jaw adductor musculature was confined to the temporal fossa and did not insert over the lateral surface of the lower jaw in non‐cynodont synapsids. An invasion of the masseteric musculature onto the zygomatic arch and the posterolateral surface of the dentary did not occur until a later, derived cynodont stage. However, several uncertainties regarding the timing and mode of muscle subdivision remained. In a similar approach, Parrington (1955) and later Crompton (1963) determined the distribution and direction of the individual jaw adductor muscles across selected cynodont taxa. In comparison to other reconstructions, these publications nevertheless remained unclear regarding the exact extent of muscle attachment sites by using simplified two‐dimensional vectors to represent individual muscles and muscle groups. Both Parrington (1955) and Crompton (1963) interpreted the majority of adductor muscles apart from the temporalis to have inserted on the post‐dentary elements in precynodont synapsids and a shift of all muscles onto the dentary in cynodonts.

Owing to the renewed interest in early mammalian evolution in the 1960s and 1970s, the adductor musculature was reconstructed or discussed for further basal synapsid, cynodont and mammaliaform taxa (e.g. Watson, 1953; Crompton & Hotton, 1967; Kemp, 1972a, 1972b, 1979; Kermack, Mussett & Rigney, 1973; Allin, 1975). However, many of the muscle reconstructions were limited to repeating existing assumptions and used a similar approach of characterising muscle forces as two‐dimensional vectors, without specifying the muscle insertions and origins anatomically. Similarly, functional aspects were mostly discussed, but rarely tested at this point. DeMar & Barghusen (1972) were among the first to use existing muscle reconstructions to test the mechanical arrangement of the lower jaw with mathematical models. Their study of moment arms and lines of muscle action applied to two‐dimensional models of different basal synapsid and cynodont taxa suggested that the development of the coronoid process was driven by selection for a higher moment arm. They further assumed that the integration of the masseter muscle in the feeding system in derived cynodonts was associated with rudimentary chewing. Using free‐body analysis and a more complex ‘bifulcral’ model, Bramble (1978) analysed jaw biomechanics across several cynodont and mammaliaform taxa. Results from this study suggested a central role of the coronoid process in the subdivision of the masseter complex, reduced stress in the delicate cynodont craniomandibular jaw joint and a contribution of the depressor muscle in stabilizing the jaw joint. While most previous biomechanical analyses assessed the vectors of muscle forces and their angle of action, the magnitude of muscle forces were not considered, or specified, in the models. Crompton & Hylander (1986) were the first to attempt to quantify muscle forces, based on approximation of activities of mandibular muscles of extant mammals. They corroborated that the synergism of the vectors and strength of mandibular adductor muscles did not contribute to the compressive joint loading for the delicate quadrate–articular jaw (Bramble, 1978), but further showed that the load‐bearing jaw joint of mammals (mammaliaforms) represents a new biomechanical framework for masticatory function, as compared to those of pre‐mammalian cynodonts. Following the approach of Crompton & Hylander (1986) mandibular function was modelled for a large range of taxa by Reed, Iriarte‐Diaz & Diekwisch (2016) using continuous variables.

In the following decades, little work was undertaken on the evolution of the mammalian jaw adductor musculature. While the discovery of fossil specimens provided new osteological data and proved that the evolution of early mammals was more complex than previously thought (e.g. Luo *et al*., 1995; Luo & Wible, 2005; Ji *et al*., 2006), myological reconstructions were rarely included in these descriptions. One of the few studies on the adductor musculature by Abdala & Damiani (2004) focussed on the differentiation and arrangement of the superficial masseter muscle within galesaurid cynodonts. Following a traditional approach of mapping potential attachment sites, the authors suggested the development of a divided masseter (into a superficial and deep component) prior to the evolution of derived cynodonts.

As for other palaeontological sub‐disciplines (Cunningham *et al*., 2014), the advent of modern computer technology and its widespread availability in the last decade has provided novel possibilities to analyse material of fossil mammals and their kin non‐destructively. Digital techniques have been used to visualise and reconstruct synapsid soft‐tissue features, such as the nasal anatomy and the olfactory apparatus (Laaß *et al*., 2011; Ruf *et al*., 2014), the endocranial anatomy (Rowe *et al*., 2011; Rodrigues, Ruf & Schultz, 2014) and inner ear morphology (Rodrigues, Ruf & Schultz, 2013; Laaß, 2015, 2016). Functional studies, involving digital visualisation, computational biomechanical approaches and three‐dimensional free body analyses have employed the vector‐based representation of adductor musculature (Gill *et al*., 2014; Jasinoski, Abdala & Fernandez, 2015; Reed *et al*., 2016). However, the potential of these techniques to reconstruct the adductor musculature digitally and in three dimensions has been unused so far. Here we employ digital technologies to assess plausible reconstructions of adductor muscle anatomy across the cynodont–mammaliaform transition. We use high‐resolution computed tomography (CT) data to render the three‐dimensional (3D) skeletal anatomy of six skulls pivotal to the debate on mammalian origins. Alongside traditional study of fossil specimens we take advantage of the ability of digital technologies to inform on the 3D spatial relationships of muscle groups and test competing hypotheses of muscle origins and insertion *via* muscle strain analysis.

## III. MATERIAL AND METHODS

### (1) Specimens, digitisation and osteological restoration

**Figure 1:**
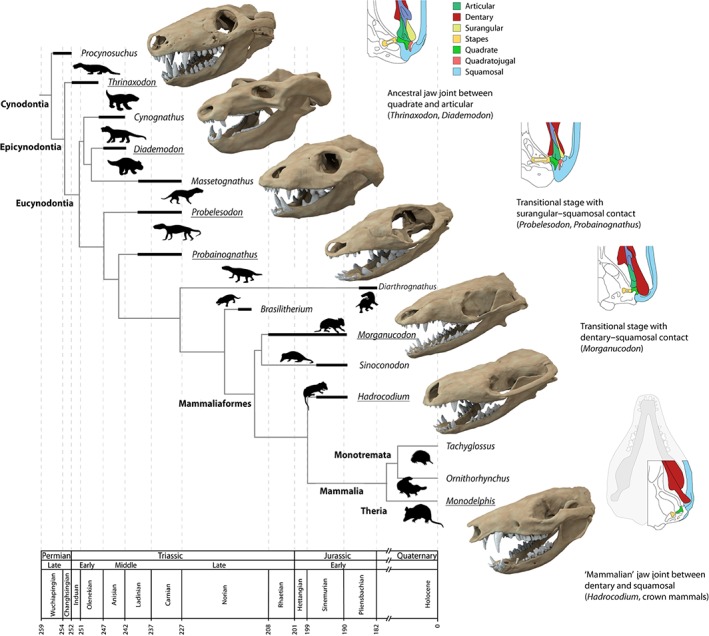
Cynodont, mammaliaform and mammalian taxa studied and discussed herein in their phylogenetic and stratigraphic context. Transitional stages of jaw joint morphology are depicted schematically on the right. Phylogeny simplified after Ruta *et al.* (2013) and Rodrigues *et al.* (2014), jaw joint schematics modified after Luo (2007). Digital skull models not to scale.

The following section provides a brief description of the specimens used in this study (Fig. 1). All specimens were digitised to create digital models. Given the preservational state of the physical specimens, digital restoration steps were required to remove preservational artefacts before the jaw adductor musculature was digitally reconstructed. For the restoration process of the different models the scan data were imported into Avizo (VSG, Visualisation Science Group, France). Data derived from CT scanning (all models except *Diademodon tetragonus*) were segmented manually to isolate fossilised bone from surrounding matrix utilising the Avizo segmentation editor. To remove taphonomic artefacts and to restore the original *in vivo* condition as closely as possible, different digital restoration steps were applied (Lautenschlager, 2016): cracks and small breaks were removed manually during the segmentation process by interpolating across the affected region. Unilaterally missing regions and elements were restored by reflecting preserved counterparts across the bilaterally symmetrical long axis of the skull. Rearticulation was performed in systematic order with the least‐deformed bones first using articulation facets and the general skull topology as a guide. Missing elements were supplemented by using information of other specimens or closely related species. Where necessary, plastic deformation was repaired by employing a landmark‐based retrodeformation approach performed with the geometric morphometrics software Landmark (version 1.6, http://www.idav.ucdavis.edu/research/EvoMorph). For that purpose corresponding, bilaterally symmetric landmarks were selected on both sides of the specimen. Based on the distance between landmarks the plane of symmetry was calculated by the software. This information was subsequently used to warp and symmetrise the model. Further model‐specific restoration steps are listed in the following sections.

#### (a) *Thrinaxodon liorhinus*

The basal, non‐mammalian cynodont *Thrinaxodon liorhinus* from the Early Triassic of Gondwana (predominantly South Africa) (Owen, 1887; Parrington, 1946) was chosen to represent the primary or ancestral condition of jaw joint evolution. CT scans of the skull (NHMUK PV R 511, see Section III.4 for Institutional abbreviations) and mandibles (NHMUK UK PV R 511a) of *Thrinaxodon liorhinus* (see online Figure S1A, B) were performed with a Nikon Metrology HMX ST 225 CT scanner set at 190 kV and 135 μA for the skull and 180 kV and 150 μA for the mandibles. The resulting 3142 projections each were processed with CT‐Pro (Nikon Metrology) reconstruction software at the Natural History Museum, London. The final image stacks (1923 × 1348 × 804 pixels, 50.2 µm voxel size for the skull; 1876 × 1661 × 433 pixels, 44.3 µm voxel size for the mandibles) were imported into Avizo (version 8, VSG) for segmentation, visualisation and further restoration and reconstruction steps.

The digital model of the skull and lower jaw of *Thrinaxodon liorhinus* required only minor restoration (see online Figure S1C–H). The specimens NHMUK PV R 511 and NHMUK PV R 511a used in this study are well preserved and nearly complete. Breaks and small fractures were removed during the segmentation process. As the left jugal is only fragmentarily preserved, the complete right element was used to produce an antimere. Both canine teeth in the skull are missing in NHMUK PV R 511 and were supplemented from a similar‐sized, second specimen (BSP 1934 VIII 506) of *Thrinaxodon liorhinus*.

#### (b) *Diademodon tetragonus*

*Diademodon tetragonus* (Seeley, 1894) represents a derived non‐mammalian cynodont commonly found in the *Cynognathus* Assemblage Zone of the South African Karoo Super Group (Rubidge, 1995) but also in the Middle Triassic of South America (Martinelli, Fuente & Abdala, 2009). Stratigraphically, it is therefore somewhat younger than *Thrinaxodon liorhinus*. In contrast to the latter, *Diademodon tetragonus* is thought to have been omnivorous or herbivorous, based on tooth morphology (Botha, Lee‐Thorp & Chinsamy, 2005) and is one of the first cynodonts to exhibit a more precise post‐canine tooth–tooth occlusion and modified jaw adductor musculature (Barghusen, 1968; Grine, 1977). The specimen used in this study (BSP 1934 VIII 17/2) was originally described as *Diademodon* (*Gomphognathus*) *grossarthi* (Broili & Schröder, 1935; Brink, 1979), now regarded as an invalid synonym of *Diademodon tetragonus* (Martinelli *et al*., 2009). The skull and lower jaw of BSP 1934 VIII 17/2 (see online Figure S2A, B) were digitised using a photogrammetry approach (Falkingham, 2012; Mallison & Wings, 2014). A Panasonic DMC‐FZ5 5‐megapixel digital camera was used to acquire 58 photographs of the skull and 24 photographs of the lower jaw. Photographs were processed with Agisoft Photoscan Standard (http://www.agisoft.ru) and the resulting 3D model exported as an .OBJ file for further processing.

The model of *Diademodon tetragonus* (see online Figure S2C–H) represents the only specimen in this study which was not digitised by CT scanning, but by using a photogrammetry approach. Although the good state of preservation and size of BSP 1934 VIII 17/2 permitted the creation of a detailed and accurate digital model, no internal features, such as the endocranial cavity, are captured by this technique. However, these details were not necessary for the identification of muscle attachment sites and the reconstruction of the jaw adductor musculature. Furthermore, as the internal details were not captured, the model required only superficial removal of breaks.

#### (c) *Probelesodon sanjuanensis*

*Probelesodon* is a derived non‐mammalian, chiniquodontid cynodont from the Late Triassic Ischigualasto Formation of Argentina (Romer, 1969*b*). It shows a number of derived, mammal‐like characters, including an elongate secondary palate, a double occipital condyle and an incipient surangular–squamosal articulation. However, it retains a functional quadrate–articular jaw joint (Romer, 1969b, 1973). *Probelesodon sanjuanensis* represents one of several species of *Probelesodon*, from which it is distinguished by the possession of a highly arched zygomatic arch and a prominent posteroventral angle on the dentary (Martinez & Forster, 1996). Abdala & Giannini (2002) considered *Probelesodon* as a junior synonym of *Chiniquodon* and regarded the individual species as different growth stages in an ontogenetic series. The same authors regard *Probelesodon sanjuanensis* as a juvenile individual. Considering the sparse fossil record for complete and unambiguous ontogenetic series of a single species, it is difficult to estimate ontogenetic effects on hard‐ and soft‐tissue reconstruction. While the overall proportions of muscle reconstructions would likely change with ontogeny, the pattern of muscle arrangement appears to be constant (Jasinoski *et al*., 2015; see also Section V).

The specimen of *Probelesodon sanjuanensis* PVSJ 411 used in this study consists of a nearly complete articulated skull and a mostly complete, attached mandible (see online Figure S3A, B). CT scans of the specimen were provided by Dr T. Rowe. The specimen was originally scanned at the University of Texas High‐Resolution X‐ray CT Facility, Austin, Texas, USA. The final image stacks (512 × 512 × 402 pixels, 200 µm voxel size) were imported into Avizo for segmentation, visualisation and further restoration and reconstruction steps.

As the specimen is largely complete and undistorted, PVSJ 411 required only little digital restoration (see online Figure S3C–H). PVSJ 411 had been partially prepared, but a large part of the internal anatomy is still embedded in calcareous matrix, which had to be removed during the segmentation process, but also obscured some fine details in the CT data set. The left squamosal is only partially preserved and was supplemented by the right squamosal. The anterior snout region has been substantially eroded. As a consequence, the premaxillary processes had to be reconstructed on the basis of comparisons with other species of *Probelesodon* (Abdala & Giannini, 2002) (see online Figure S3C, E).

#### (d) *Probainognathus jenseni*

*Probainognathus jenseni* belongs to a group of derived, carnivorous non‐mammalian cynodonts from the Late Triassic of Argentina (Romer, 1970). It is distinguished from more basal taxa, such as *Thrinaxodon liorhinus* and *Diademodon tetragonus*, by an incipient contact between the surangular and the squamosal. The specimen used in this study (PVSJ 410) represents a juvenile individual referred to as *Probainognathus* sp. (Bonaparte & Crompton, 1994), which shows more pronounced mammal‐like characters compared to mature specimens, including a relatively larger braincase, a slender zygomatic arch, and postcanine teeth morphologically similar to those of *Morganucodon* (Kemp, 2005). However, it should be noted that the juvenile status of this specimen has precluded a precise taxonomic identification. A preliminary phylogenetic analysis has suggested the specimen to be an unnamed and unspecified probainognathian cynodont more derived than *Probainognathus* (Fernandez *et al*., 2011). Although the taxonomic position has no influence on the reconstructed morphology of the jaw adductor complex in this study, caution is necessary regarding evolutionary hypotheses. Comparisons with adult and unambiguously identified specimens of *Probainognathus* have therefore been made.

CT scans of the skull and articulated mandibles of PVSJ 410 (see online Figure S4A, B) were provided by Dr T. Rowe. The specimen was originally scanned at the University of Texas High‐Resolution X‐ray CT Facility. The final image stacks (512 × 512 × 496 pixels, 85 µm voxel size) were imported into Avizo for segmentation, visualisation and further restoration and reconstruction steps.

PVSJ 410 consists of an articulated skull and lower jaw and is largely complete, except that parts of the left side, including the jugal, postorbital, squamosal, quadrate and most of the post‐dentary region are missing (see online Figure S4A, B). The cranial restoration was hence primarily based on the complete right side. The right postorbital had been displaced anteriorly and ventrally and had to be rearticulated according to the articulation facets on the jugal and the general dimensions of the postorbital region. Although the specimen had been prepared externally, parts of the braincase and the narial and palatal region were still embedded in matrix. Using the CT data is was possible to remove the matrix digitally. However, due to the preservation and low scan resolution, not all the morphology of the affected regions could be restored, resulting in some gaps in the palate and the anterolateral braincase wall. The gaps were closed manually by connecting the adjacent bone surfaces (see online Figure S4C–H).

#### (e) *Morganucodon oehleri*

*Morganucodon* (Kühne, 1949) is a basal mammaliaform found in Late Triassic and Early Jurassic deposits of Europe, China and North America. The species *Morganucodon oehleri* is represented by largely complete and articulated cranial skeletons from the Early Jurassic of the Lower Lufeng Formation of Yunnan in China (Kermack *et al*., 1973; Kermack, Mussett & Rigney, 1981; Luo *et al*., 1995). The taxon presents a further transitional stage in jaw joint evolution by possessing a ball‐like dentary condyle articulating with a defined glenoid cavity in the squamosal alongside an articular–quadrate joint. For this study, an articulated skull and mandible (holotype FMNH CUP 2320) were CT scanned at the University of Chicago using a General Electrics v|tome|x s 240 scanner. Scan parameters were set at 110 kV and 80 μA.

A second specimen (IVPP 8685) was scanned at the University of Texas High‐Resolution X‐ray CT Facility using a XYZ scanner with parameters set at 150 kV and 38 μA. (Final image stacks for FMNH CUP 2320: 1600 × 1600 × 3576 pixels, 10.7 µm voxel size; IVPP 8685: 1024 × 1024 × 960, 25.7 µm voxel size.) Additional disarticulated elements of *Morganucodon watsoni* (NHMUK PV M 26144, articulated squamosal and petrosal; NHMUK PV M 92838 and M 92843, isolated quadrates; NHMUK PV M 27410, isolated fragmentary jugal) were scanned at the University of Bristol using a Bruker SkyScan1272 Micro‐CT scanner with parameters set at 166 kV and 60 μA. The final image stacks (2452 × 2452 × 1106 pixels, 5.1 µm voxel size) were imported into Avizo for segmentation, visualisation and further restoration and reconstruction steps.

FMNH CUP 2320 represents the most complete and best preserved specimen for any *Morganucodon* species and was used mainly for the restoration process (see online Figure S5A, B). Although it consists of an articulated and mostly complete skull and isolated right mandible, the specimen has suffered considerably from taphonomic processes and erosion. The left side of the specimen has been eroded more substantially, therefore the restoration focussed to a large extent on the more completely preserved right side. The individual elements were separately segmented using Avizo. Small breaks and cracks were removed during the segmentation process by interpolating along the complete bone margins. The left premaxilla, the right maxilla, the right lacrimal and the right frontal, post repair, were mirrored across the bilaterally symmetrical long axis of the skull to produce their antimere. The proportions of each element and of the completed skull and mandible models were compared throughout to those of IVPP 8685 and pre‐existing reconstructions to ensure consistency. As neither FMNH CUP 2320 nor IVPP 8685 preserve the squamosal, the quadrates and the jugals, these elements were supplemented by isolated specimens of *Morganucodon watsoni* (NHMUK PV M 26144, articulated squamosal and petrosal; NHMUK PV M 92838 and M 92843, isolated quadrates; NHMUK PV M 27410, isolated fragmentary jugal). All elements were rearticulated using Avizo. As the jugal is only partially preserved in any known specimen of *Morganucodon*, parts of the zygomatic arch had to be interpolated between the fragmentary anterior portion of the jugal and the squamosal. This step was performed manually with the preserved elements as a guide. The curvature of the zygomatic arch was modelled so that it created a natural outline without bowing too much laterally or medially (see online Figure S5C–H). The restored model differs from previous restorations (Kermack *et al*., 1981) in the lateral expansion of the jugal. This is due to a distinct kink in the jugal, which appears to be natural, as several specimens show this morphology, while the CT data revealed no evidence for taphonomic artefacts (i.e. breaks, bending). Similarly, the orbitosphenoid region between the frontal, lacrimal and the braincase elements had to be reconstructed, as this region is not preserved fully. The reconstruction was based on the topological constraints provided by the surrounding and articulating bones and published reconstructions (Kermack *et al*., 1981). The left mandible is largely complete and had been removed from the articulated skull during preparation. Missing regions, such as the dorsal portion of the coronoid process were supplemented from IVPP 8685.

#### (f) *Hadrocodium wui*

*Hadrocodium wui* (Luo *et al*., 2001) is a basal mammaliaform from the Lower Jurassic (Sinemurian) of China. The enlarged cranial cavity, lack of a postdentary trough and most importantly the fully developed jaw articulation between the dentary and the squamosal make *Hadrocodium wui* more derived than *Morganucodon*. *Hadrocodium wui* is represented by a single specimen (IVPP 8275) consisting of a nearly complete skull and articulated lower jaw. The specimen was scanned at the University of Texas High‐Resolution X‐ray CT Facility using a XYZ scanner with parameters set at 150 kV and 38 μA. The final image stacks (1024 × 1024 × 735 pixels, 19 µm voxel size) were imported into Avizo for segmentation, visualisation and further restoration and reconstruction steps.

The specimen used in this study is relatively complete and consists of an articulated skull, and most of the lower jaws. However, the specimen has suffered considerably from taphonomic deformation, which caused the skull roof around the parietals and frontals to collapse inwards. For the restoration process, the individual elements were segmented separately and breaks and cracks were removed using Avizo. The rearticulation of the individual elements was performed in a systematic order with the elements and regions affected least by taphonomy articulated first. This allowed a rearticulation of the displaced frontal and parietal bones, which were rotated and translated in Avizo to create a consistent morphology of the skull roof (see online Figure S6A, B).

Due to erosion and other taphonomic processes, IVPP 8275 is missing parts of the zygomatic arch and of the narial process of the premaxilla. Similar to the restoration process of *Morganucodon*, the regions were interpolated manually as no other specimen of *Hadrocodium wui* exists. The zygomatic arch was reconstructed between the anterior portion of the jugal and the squamosal with a minimum of curvature to create a natural outline. The same principle was applied for the narial process of the premaxilla (see online Figure S6C–H). Similar to the condition found in *Morganucodon*, the orbitosphenoid is not preserved and had to be reconstructed in *Hadrocodium wui* on the basis of topological criteria and published reconstructions (Luo *et al*., 2001).

#### (g) *Monodelphis domestica*

*Monodelphis domestica* (Gray short‐tailed opossum) was chosen as an extant representative and comparative specimen for the osteology and jaw adductor morphology. *Monodelphis* represents the fully mammalian stage of jaw joint evolution and it (or alternatively the closely related *Didelphis*) has been used as an extant standard in previous studies on the evolution of the jaw adductor complex (e.g. Crompton, 1995; Abdala & Damiani, 2004; Luo, 2011). For this study, a frozen, but otherwise intact specimen of *Monodelphis domestica* was obtained on loan from the National Museum of Scotland, Edinburgh. The specimen was CT scanned twice, without and with a contrast‐enhancing agent to improve soft‐tissue resolution. Both scans were performed at the μ‐VIS facility of the University of Southampton using a Nikon Metrology HMX ST 225 CT scanner with parameters set at 150 kV and 60 μA. The specimen was defrosted thoroughly before being scanned without any staining agent. Before the second scan, the specimen was submerged in a 10% solution of I_2_KI in 4% paraformaldehyde in phosphate‐buffered saline, and stored in a refrigerator. After 16 days, the specimen was removed from the iodine solution and the CT scan was repeated with the same settings. The final image stacks (2000 × 2000 × 800 pixels, 33 µm voxel size) were imported into Avizo for segmentation, visualisation and further restoration and reconstruction steps.

### (2) Myological reconstructions

**Figure 2:**
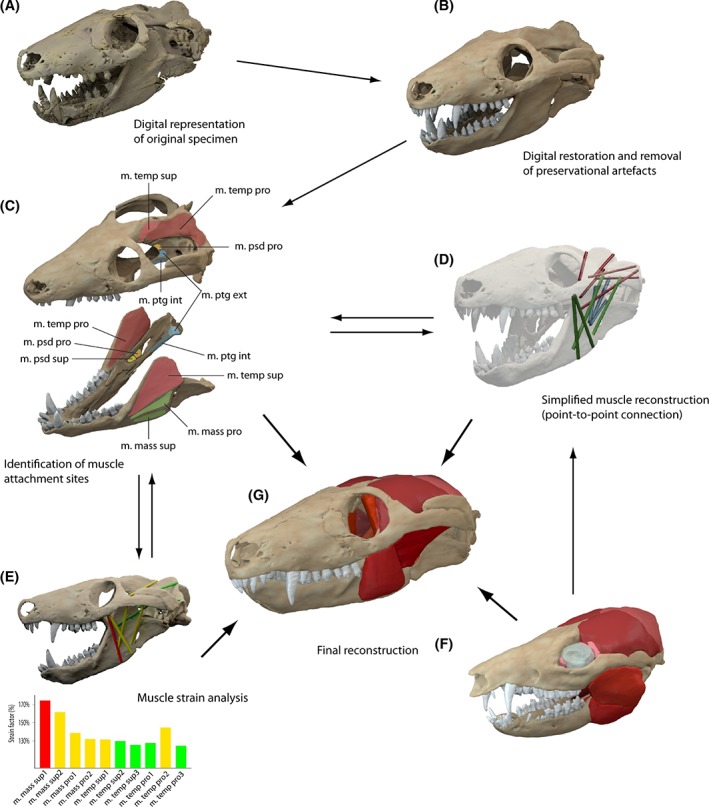
Flow chart illustrating the digital reconstruction process of the jaw adductor musculature applied in this study. (A) Digital representation of fossil specimen; (B) restoration of osteology; (C) identification of muscle origins and insertions based on osteological correlates on actual specimen and/or visible *via* digital reconstruction; (D) simplified muscle reconstruction (‘cylinder model’) using point‐to‐point connections of corresponding muscle attachments (Lautenschlager, 2013); (E) analysis of muscle strain capabilities (see Lautenschlager, 2015); (F) comparisons with extant taxa, which are phylogenetically closely related or form an extant phylogenetic bracket; (G) final muscle reconstruction. See Section III.4 for muscle abbreviations.

The muscle anatomy of the studied taxa was reconstructed digitally following a protocol laid out by Lautenschlager (2013): muscle origin and insertion sites were identified on both the physical and digital specimens based on osteological correlates and obvious surface features such as muscle scars, depressions, ridges, crests or other bony protrusions (Fig. 2C). Where exact locations could not be identified, topological criteria were applied, i.e. clearly identified muscle sites constrained the location of an adjacent muscle. Following their identification on the skull and the mandible, corresponding attachment sites were connected by simple point‐to‐point connections (‘muscle tubes’) using Avizo (Fig. 2D). Depending on the size of the muscle attachments, up to 10 muscle tubes were used to cover the maximum extent of the respective muscle. When all muscles were provisionally modelled in this way, the arrangement and topology was inspected for intersections with other muscles or the bony structure. In such cases, the arrangement was modified until all muscle tubes were accommodated without intersections. In a final step, the diameter of the muscle tubes was increased uniformly in Avizo until two or more muscle tubes of the same muscle merged together, until a different muscle was encountered or until a bony structure was reached. Where possible, neurovascular criteria such as the location of branches of the trigeminal nerve were used to distinguish separate muscle groups. As a further constraint the eyeball was modelled as a simple sphere with a diameter that permitted a tight fit into the orbital cavity. During the reconstruction process, the model of *Monodelphis domestica* derived from contrast‐enhanced CT scanning was used for comparison (Fig. 2F). Interactive 3D PDF files (see online Figure S7–S13) were created for all taxa following the protocol outlined in Lautenschlager (2014).

### (3) Muscle strain and gape analysis

To test the hypothesised muscle reconstructions, a further approach taking muscle strain into account was applied (Lautenschlager, 2015) (Fig. 2E). For this purpose, models were imported into the freely available 3D visualisation and animation software Blender (version 2.71, http://www.blender.org) as .PLY files. Model sizes were kept at typically 250000 elements for the skulls and 100000 elements for the lower jaws. For each taxon, the skull and lower jaw were treated as separate components, with the centre of rotation for the lower jaw positioned at the level of the jaw joint. For the strain analyses, an opening movement of the lower jaw models was simulated. The degree of rotation representing the gape angle was set manually to range from 0 to 50 degrees in 0.5 degree steps to capture all necessary jaw positions and corresponding muscle strain values.

The jaw adductor muscles for the different taxa were modelled as cylinders selected from the in‐built geometry primitives library and positioned to connect corresponding muscle origin and insertion sites. A two‐element armature originating from the jaw joint was created to allow deformation of the muscle cylinders. The individual components of the armature represent the skull (kept immobile) and the mandible (mobile) and were connected to the muscle cylinders. For the duration of the opening cycle, length changes between the muscle cylinders in both the relaxed and stretched state (= strain ratio) were recorded. Additionally, the muscle cylinders were colour‐coded to change colour when a certain strain ratio was exceeded (green: <130%, yellow: 130–170%, red: >170%). Values were chosen to represent the optimal tetanic tension (100–130%), when maximal muscular contraction can be achieved, and maximal tetanic tension limit (170%), above which contraction is no longer possible (Nigg & Herzog, 2007; Sherwood, Klandorf & Yancey, 2012).

### (4) Anatomical and institutional abbreviations

m. mass pro, m. masseter pars profunda; m. mass sup, m. masseter pars superficialis; m. mass sup ext, m. masseter pars superficialis external body; m. mass sup int, m. masseter pars superficialis internal body; m. oc, ocular muscles; m. psd pro, m. pseudotemporalis pars profunda; m. psd sup, m. pseudotemporalis pars superficialis; m. ptg ext, m. pterygoideus externus; m. ptg int, m. pterygoideus internus; m. temp pro, m. temporalis pars profunda; m. temp sup, m. temporalis pars superficialis; V_2_, foramen for maxillary branch of trigeminal nerve.

BSP, Bayerische Staatssammlung für Historische Geologie und Paläontologie, Munich, Germany; FMNH CUP, Field Museum of Natural History, Chicago, USA; IVPP, Institute for Vertebrate Palaeontology and Palaeoanthropology, Beijing, China; NHMUK PV, Natural History Museum, London, UK; PVSJ, Museo de Ciencias Naturales, Universidad Nacional de San Juan, Argentina.

## IV. MORPHOLOGY OF THE ADDUCTOR COMPLEX

### (1) Extant condition: *Monodelphis domestica*

The morphology of the jaw adductor muscles in *Monodelphis domestica* is described here first to provide a baseline for comparison with the fossil taxa in subsequent sections. The description is based on a digital dissection using contrast‐enhanced CT scanning and digital visualisation tools to determine the different jaw muscle components and their arrangement. *Monodelphis* and the the closely related genus *Didelphis* retain a number of basal marsupial features in the masticatory apparatus (e.g unmodified incisors, dilambdodont upper molars) and their muscle arrangement has been regarded closest to the ancestral condition by several authors (e.g. Adams, 1918; Barghusen, 1968; Hiiemae & Jenkins, 1969; Crompton, 1995; Abdala & Damiani, 2004; Luo, 2011). The jaw adductor complex of *Monodelphis domestica* consists of the m. temporalis, the m. masseter and the m. pterygoideus, including further subdivisions within each of these muscles. The terminology follows the commonly used classification for mammalian cranial myology (Druzinsky, Doherty & De Vree, 2011).

**Figure 3:**
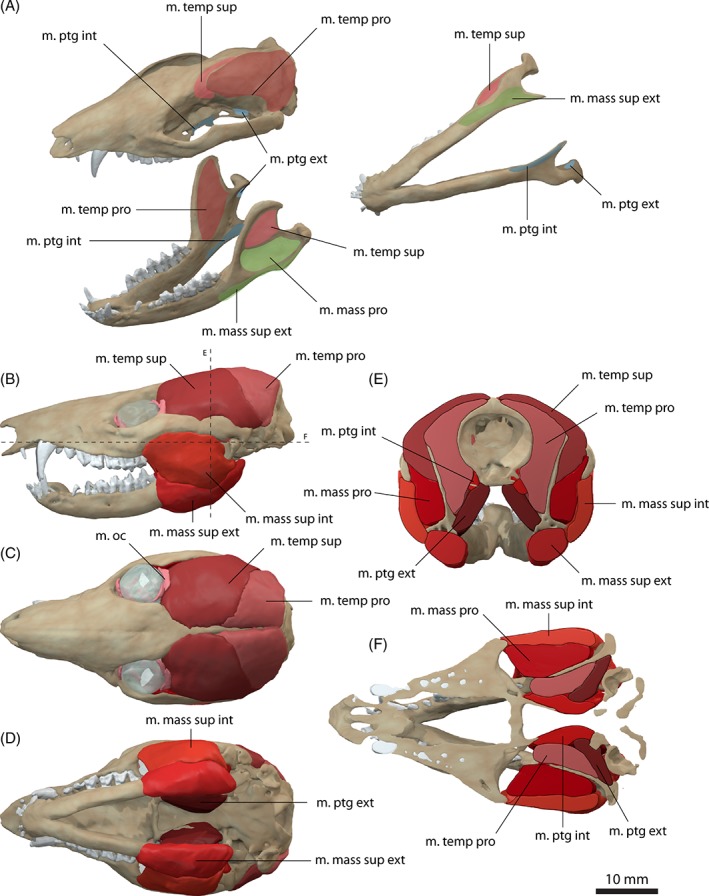
Jaw adductor musculature of *Monodelphis domestica*. (A) Muscle origins and insertions on the skull and mandible. Muscle arrangement in (B) left lateral, (C) dorsal and (D) ventral view, (E) coronal and (F) horizontal section through the skull. See Section III.4 for muscle abbreviations.

The m. temporalis forms the largest muscle complex in *Monodelphis domestica*, occupying nearly the entire temporal region. It bulges slightly dorsally and laterally to the skull, but is otherwise flush with the margins of the sagittal and occipital crests and the zygomatic arch (Fig. 3B, E), similar to the condition observed in *Didelphis marsupialis* (Turnbull, 1970). The m. temporalis originates from the lateral braincase wall and the surface of the parietal and ensheaths the coronoid process of the dentary (Fig. 3A, E). In *Monodelphis domestica*, the m. temporalis is subdivided into a superficial (pars superficialis) and a deep portion (pars profunda). The m. temporalis pars superficialis originates anterior to the pars profunda from the anteriormost part of the parietal and posterior surface of the frontal (Fig. 3A). Anteriorly, the muscle extends up to a distinct vertical ridge on the lateral surface of the frontal (equivalent to the inferior temporal line in human anatomy). It runs across the anterior half of the pars profunda and inserts on the dorsal half of the lateral surface of the coronoid process on the dentary (Fig. 3A, E). The m. temporalis pars profunda lies deep and posteriorly to the pars superficialis and has a fleshy origin from the lateral surface of the parietal and the anteromedial surface of the squamosal (Fig. 3A, E). The pars profunda is considerably larger than its superficial counterpart. Ventrally, the muscle attachment extends approximately to the suture of the parietal with the alisphenoid and the squamosal. The m. temporalis pars profunda inserts along the medial surface of the coronoid process on the dentary (Fig. 3A). The pars profunda and superficialis meet anterior to the coronoid process and have a similar extension. On the skull, the division between both parts of the m. temporalis is located approximately along the parietal/frontal suture. The arrangement and terminology of the subdivision of the m. temporalis observed in *Monodelphis domestica* generally corresponds well to that of Turnbull (1970) for *Didelphis marsupialis*. In comparison, Hiiemae & Jenkins (1969) did not homologise the temporal adductor muscles in their study and simply referred to them as internal and posterior adductor (partially equivalent to the m. temporalis pars profunda) and external adductor (partially equivalent to the m. temporalis superficialis) in reference to their position. In *Didelphis marsupialis*, the muscle fibres of the m. temporalis have been described as fanning out across the lateral surface of the parietal and frontal and converging towards the coronoid process (Turnbull, 1970). As far as the resolution of the CT data set permits, this non‐uniform pattern of fibre orientation is also observed in *Monodelphis domestica*.

The m. masseter occupies the region between the zygomatic arch and the mandible and a subdivision into a superficial (pars superficialis) and a deep part (pars profunda) can be observed in *Monodelphis domestica* (Fig. 3). The m. masseter pars superficialis originates from the anteriormost region of the zygomatic arch along the jugal/maxilla suture (Fig. 3A, E). The attachment is located at the level of the last maxillary tooth ventral to the orbit/eyeball. The respective region of the zygomatic arch shows a distinct angulation, which has been termed suborbital angulation by Abdala & Damiani (2004) and has been regarded as an osteological correlate for the subdivision of the m. masseter and the attachment of the pars superficialis. The m. masseter pars superficialis inserts along the ventrolateral surface of the dentary ventral to the fossa, into which the deep counterpart attaches (Fig. 3A). On the ventral surface, the muscle attachment extends onto the inflected angular process. Anteriorly, the pars superficialis reaches the level of the last dentary tooth. As can be observed in the contrast‐enhanced CT data set, the pars superficialis consists of an external and internal body. The internal body occupies the dorsal half of the muscle; the external body occupies the ventral half along the insertion of the muscle. This subdivision of the pars superficialis has not been observed in *Didelphis marsupialis* (Hiiemae & Jenkins, 1969; Turnbull, 1970), but is present in other marsupials (Sharp & Trusler, 2015).

The m. masseter pars profunda is nearly completely covered by the pars superficialis (Fig. 3E). It originates from the ventral and medial surfaces of the zygomatic arch and extends along the entire length of the zygomatic arch (Fig. 3A). The pars profunda inserts into a depression ventral to the attachment of the m. temporalis superficialis. A third subdivision of the m. masseter, the m. zygomatico‐mandibularis, described in *Didelphis marsupialis* (Turnbull, 1970) and other taxa (Druzinsky *et al*., 2011), cannot be recognised in *Monodelphis domestica*, but this is likely due to insufficient resolution of the CT data set.

The m. pterygoideus is also subdivided in *Monodelphis domestica*. The m. pterygoideus internus originates from the ventrolateral surface of the pterygoid (Fig. 3A, E). It is short and compact and inserts along the medioventral surface of the dentary and along the inflected angular process. Anteriorly, the attachment extends to the level of the m. temporalis profunda, just posterior to the last dentary tooth. The m. pterygoideus externus is largely covered by the m. pterygoideus internus in ventral aspect and is considerably smaller than the latter. It originates dorsal to the attachment of the m. pterygoideus internus from the lateral surface of the alisphenoid (Fig. 3A, E). On the dentary, it inserts into a shallow depression on the medial surface of the condylar process. The arrangement and subdivision of the m. pterygoideus in *Monodelphis domestica* corresponds to the condition found in *Didelphis marsupialis* (Hiiemae & Jenkins, 1969; Turnbull, 1970).

### (2) *Thrinaxodon liorhinus*

**Figure 4:**
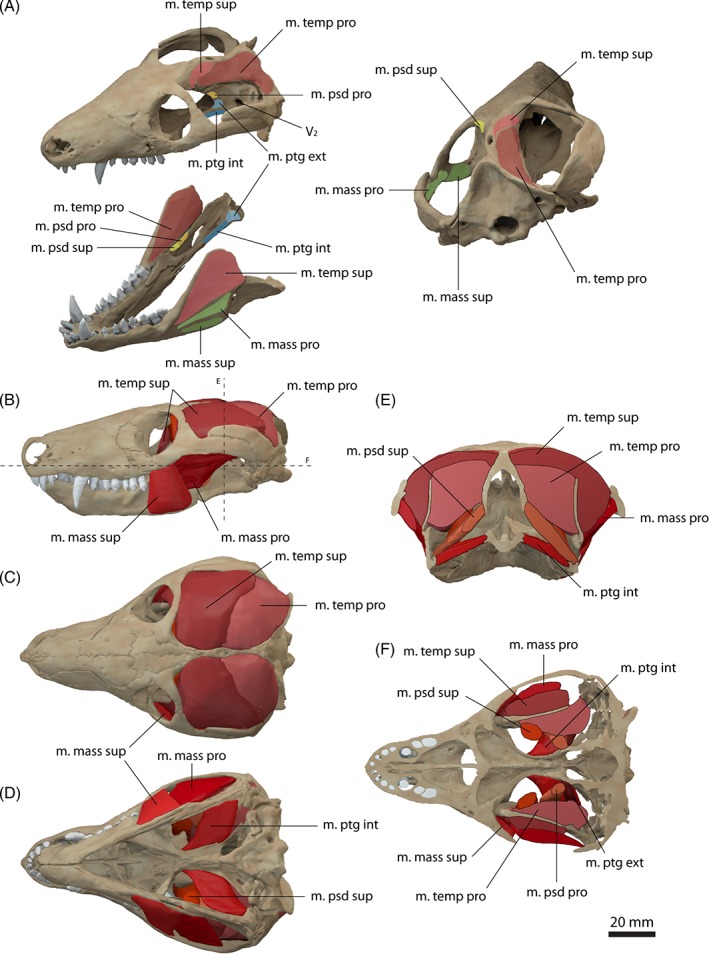
Jaw adductor musculature of *Thrinaxodon liorhinus* *.* (A) Muscle origins and insertions on the skull and mandible. Muscle arrangement in (B) left lateral, (C) dorsal and (D) ventral view, (E) coronal and (F) horizontal section through the skull. See Section III.4 for muscle abbreviations.

It is generally agreed that a mammal‐like muscle division, comprising a temporalis, a masseter and a pterygoideus muscle group, was fully present in derived cynodonts, such as *Cynognathus* and *Diademodon* (Watson, 1948; Crompton, 1963; Barghusen, 1968). However, the evolutionary origins of this subdivision possibly date back to more basal galesaurid cynodonts (Abdala & Damiani, 2004). By contrast, basal therapsids retained the ancestral condition, in which the m. adductor mandibulae externus and the m. adductor mandibulae internus muscle groups formed the major components of the jaw adductor complex (Barghusen, 1968). A differentiation and transformation of the masticatory muscles appears to take place in Theriodontia following the development of an increase in size of the temporal opening in the skull, the modification of the mandible and the subsequent transfer of the temporalis muscle group from the postdentary bones to the dentary (Bramble, 1978; Abdala & Damiani, 2004). Consequently, the basal cynodont *Thrinaxodon liorhinus* exhibits a mammalian‐like pattern of muscle division, from which further modifications were derived. The arrangement and general subdivision of the jaw adductor musculature of *Thrinaxodon liorhinus* corresponds largely to the mammalian condition. The adductor complex comprises a m. temporalis, a m. masseter, a m. pterygoideus, but possibly also retains a m. pseudotemporalis, including further subdivisions of each muscle group (Fig. 4).

**Figure 5:**
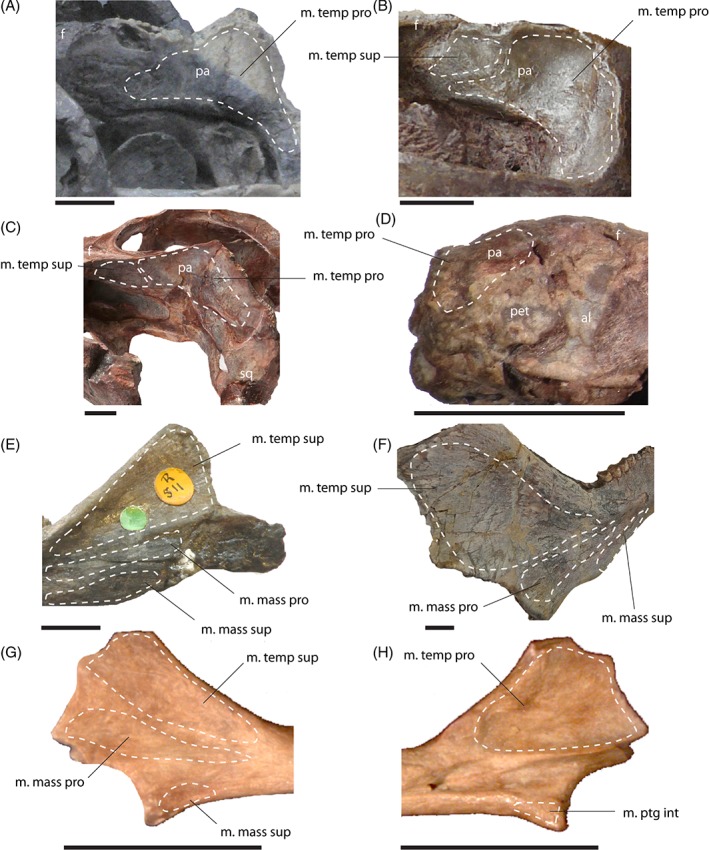
Osteological correlates of the jaw adductor musculature in the skull (A–D) and lower jaw (E–H). Temporal region of (A) *Thrinaxodon liorhinus* (NHMUK PV R 511), (B) *Thrinaxodon liorhinus* (BSP 1934 VIII 506) and (C) *Diademodon tetragonus* (BSP 1934 VIII 17/2) in left lateral view. (D) *Morganucodon oehleri* (FMNH CUP 2320) in right lateral view. Mandible of (E) *Thrinaxodon liorhinus* (NHMUK PV R 511) in lateral view. (F) *Diademodon tetragonus* (BSP 1934 VIII 17/2) in lateral view. (G) *Morganucodon watsoni* (NHMUK PV M 27252) in lateral and (H) medial view. Scale bars, 10 mm. See Section III.4 for muscle abbreviations; al, alisphenoid; f, frontal; pa, parietal; pet, petrosal; sq, squamosal.

The m. temporalis is most likely subdivided into a superficial and a deep component as indicated by separate insertions on the elevated coronoid process of the dentary (Barghusen, 1968, 1972). The cranial origin of the m. temporalis pars superficialis is only weakly constrained. A distinct temporal line, as present in *Monodelphis domestica* is absent in *Thrinaxodon liorhinus*. The expansion of the braincase along the suture with the postorbital could indicate the anterior extent of the muscle attachment which was reconstructed accordingly here. Barghusen (1968, 1972) suggested an additional aponeurotic origin of the temporalis muscle, evident by the sharp edges along the sagittal and occipital crests. On the dentary, the m. temporalis pars superficialis inserts on the lateral surface of the coronoid process onto the dorsal portion of a well‐developed fossa (Figs 4A and 5E). Ventrally, the insertion site is bounded by a prominent ridge. The bony attachment of the m. temporalis pars profunda is marked by an elongate depression on the lateral surface of the parietal and the anterior surface of the squamosal (Figs 4A and 5A, B). The m. temporalis pars profunda inserts on the medial surface of the coronoid process of the dentary. A faint ridge dorsal to the contact of the dentary with the surangular indicates the ventral extent of the attachment. As in *Monodelphis domestica*, the m. temporalis was reconstructed to be flush with the margins of the sagittal and occipital crests and the zygomatic arch.

While most authors agreed on the differentiation of the m. temporalis and the m. masseter (e.g. Barghusen, 1968; Bramble, 1978), the m. masseter and its possible subdivision into a superficial and a deep component in non‐mammaliaform cynodonts has been the focus of debate in recent decades. Barghusen (1968, 1972, 1973) and DeMar & Barghusen (1972) suggested the presence of an undivided m. masseter (equivalent to the m. masseter pars profunda) in *Thrinaxodon liorhinus* and other basal cynodonts based on the presumed lack of osteological correlates. Rather, these authors suggested a developing stage of the m. masseter in basal cynodonts, with a fully differentiated m. masseter in probainognathids and more‐derived eucynodonts. By contrast, Allin & Hopson (1992) and later Abdala & Damiani (2004) argued for differentiation of the m. masseter near the base of Epicynodontia. They regarded the angulation of the zygomatic arch as an indication for a separate origin of the m. masseter pars superficialis. A similar angulation, from which a separate m. masseter pars superficialis originates is present in didelphid marsupials (Abdala & Damiani, 2004). Furthermore, the position of the angulation in homologous to the suborbital process of cynognathian cynodonts (e.g. *Trirachodon*, *Diademodon*). Although only weakly developed in *Thrinaxodon liorhinus*, such a suborbital angulation is present in the more basal galesaurid cynodont *Galesaurus* as well as in derived epicynodonts, such as *Platycraniellus* (Abdala & Damiani, 2004). Consequently, the most parsimonious assumption for the differentiation of the m. masseter would place it at the common ancestor of both clades, and close to the base of Epicynodontia, thereby including *Thrinaxodon liorhinus*. A differentiated m. masseter, subdivided into a superficial (pars superficialis) and a deep part (pars profunda) was therefore reconstructed here for *Thrinaxodon liorhinus*. A separate muscle body representing the m. zygomaticomandibularis was not reconstructed due to the lack of unambiguous osteological correlates and the difficulty in differentiating this muscle in extant taxa (Bramble, 1978).

**Figure 6:**
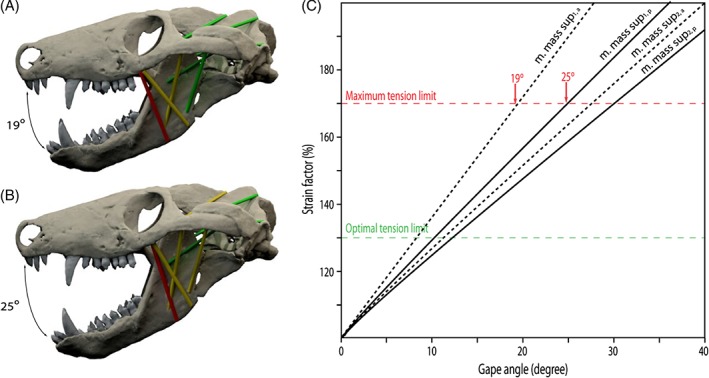
Strain analysis for different muscle positions of the m. masseter pars superficialis in *Thrinaxodon liorhinus*. (A) Anterior cranial attachment close to the maxilla/jugal suture. (B) Posterior attachment of the m. masseter pars superficialis resulting in different possible gape angles. (C) Muscle strain plotted against gape angle for different muscle attachments. Subscripts a (anterior) and p (posterior) indicate muscle position. Colour coding indicates strain ratios below 130% (green), between 130 and 170% (yellow) and over 170% (red). See Section III.4 for muscle abbreviations.

The m. masseter pars superficialis originates from the ventromedial surface of the anteriormost part of the zygomatic arch near the maxilla/jugal suture (Fig. 4A, B). The exact extent of the attachment between the maxilla contact anteriorly and the postorbital bar posteriorly is difficult to determine. Abdala & Damiani (2004) reconstructed a small attachment site on the zygomatic process of the maxilla. However, such a forward position of the cranial attachment would result in interference with the posteriormost tooth in *Thrinaxodon liorhinus*. Furthermore, analysis of the muscle strain demonstrates that an anterior attachment of the m. masseter pars superficialis would only permit small gape angles, which would barely clear the upper and lower canines (Fig. 6). Therefore, the m. masseter pars superficialis was reconstructed here with an origin somewhat posterior to the maxilla/jugal suture extending up to the level of the postorbital process. The muscle inserts on the posteroventral edge of the dentary (Figs 4A, B, D and 5E). A dentary angle is only weakly developed in *Thrinaxodon liorhinus*, but the respective region is homologous to the angular process of crown‐mammals (Luo, Kielan‐Jaworowska & Cifelli, 2002) and thereby also to the attachment site of the m. masseter superficialis in extant mammals. The m. masseter pars profunda has its origin along the medial surface of the zygomatic arch, consistent with previous reconstructions (e.g. Watson, 1948; Crompton, 1963; Bramble, 1978). The attachment extends from the level of the postorbital process to the squamosal and is indicated by a shallow depression (Fig. 4A, E). On the dentary, the m. masseter pars profunda inserts on the fossa ventral to the attachment of the m. temporalis pars superficialis (Figs 4A and 5E). The insertion is marked by a near‐vertical ridge separating both muscle components. An extension of the m. masseter pars profunda (but also of the m. temporalis pars superficialis) onto the postdentary bones has generally been regarded as unlikely due to the presence of simple patent sutures (Watson, 1948, 1953; Barghusen, 1968; Bramble, 1978). Because the dentary and postdentary elements are not sutured or rigidly articulated with each other, the loose contact between the dentary and postdentary elements would not only make it ineffective for the forces from muscles attached to the postdentary elements to be transmitted onto the tooth‐bearing dentary, but also risk detachment of postdentary bones with muscle contraction. As in *Monodelphis domestica*, the m. pterygoideus is most likely subdivided in *Thrinaxodon liorhinus* into an internal (often termed anterior or medial) and an external (often termed lateral) part. The m. pterygoideus externus arises from the ventral portion of the lateral surface of the epipterygoid (Fig. 4A). This attachment is indicated by a moderate depression bounded by a posterior ridge and is homologous with the origin of the reptilian m. pterygoideus dorsalis from the epipterygoid in more basal synapsids (Watson, 1953; Barry, 1965). The m. pterygoideus externus inserts on the medial surface of the articular, between the articular condyle and the attachment of the m. pterygoideus internus (Fig. 4A), analogous to the attachment found in *Monodelphis domestica*. The m. pterygoideus internus originates from the ventrolateral surface of the pterygoid (Fig. 4A, D). The respective area is deeply notched between the contact with the palate anteriorly and the alisphenoid posteriorly. For the mandibular insertion, a number of different attachments have been discussed in the past. Crompton (1963), and later others (e.g. Kemp, 1972*a*), suggested an attachment to the dentary angle in cynodonts. By contrast, other workers (e.g. Watson, 1953; Barghusen, 1968; Allin, 1975) favoured an attachment to the medial, ventral and partially lateral surfaces of the angular, wrapping ventrally around the bone (similar to the reptilian m. pterygoideus ventralis). Considering the relatively large size of the postdentary bones, it seems more plausible that the repositioning of the m. pterygoideus internus to the dentary had not yet taken place in *Thrinaxodon liorhinus*. Bramble (1978) argued that muscle scars on the dentary angle may relate to the attachment of a digastric muscle rather than the m. pterygoideus. Therefore, the m. pterygoideus has been reconstructed to insert on the medioventral surface of the angular here (Fig. 4A, D). An extension of the muscle onto the lateral surface, as suggested by some authors (Watson, 1953; Allin, 1975), is possible, but osteological correlates are not clear enough to support this unambiguously.

In addition to the mammal‐like adductor muscles, it is possible that *Thrinaxodon liorhinus* retained the m. pseudotemporalis muscle group, typically found in archosaurs and more basal synapsids (Barghusen, 1973; Holliday & Witmer, 2007). In these taxa, the pseudotemporalis fills the anterior portion of the temporal fossa and is associated with the postorbital, epipterygoid and parietal bones. The m. pseudotemporalis is subdivided into a superficial and deep component. In *Thrinaxodon liorhinus*, osteological correlates in the form of a shallow depression on the anteromedial surface of the adductor chamber and sharp margins of the postorbital, indicate that the m. pseudotemporalis superficialis could originate from the lateroventral surface of the postorbital (Fig. 4A). We interpret that the m. pseudotemporalis superficialis would insert on the mandible between the dentary and the post‐dentary bones in *Thrinaxodon liorhinus*. Overall, this adductor muscle would be attached to both the dentary and the postdentary bones. The narrow gap between the respective bones indicates only a weak bony attachment of the muscle, or more likely an attachment *via* a tendon sheet together with the m. pseudotemporalis profundus, as interpreted for basal synapsids by Barghusen (1973). The m. pseudotemporalis profundus in turn originates from the epipterygoid, dorsal to the attachment of the m. pterygoideus externus (Fig. 4A). The topology of the neurovascular foramina further would support the presence of a pseudotemporalis group in *Thrinaxodon liorhinus*. In the reptilian condition the m. adductor mandibulae internus group (which includes the m. pseudotemporalis and the m. pterygoideus) is separated from the m. adductor mandibulae externus group (which gives rise to the mammalian m. temporalis and m. masseter) by the maxillary branch of the trigeminal nerve. As reconstructed here, the bony foramen for the trigeminal nerve is located approximately between the m. pseudotemporalis/m. pterygoideus and the m. temporalis/m. masseter divisions (Fig. 4A).

### (3) *Diademodon tetragonus*

**Figure 7:**
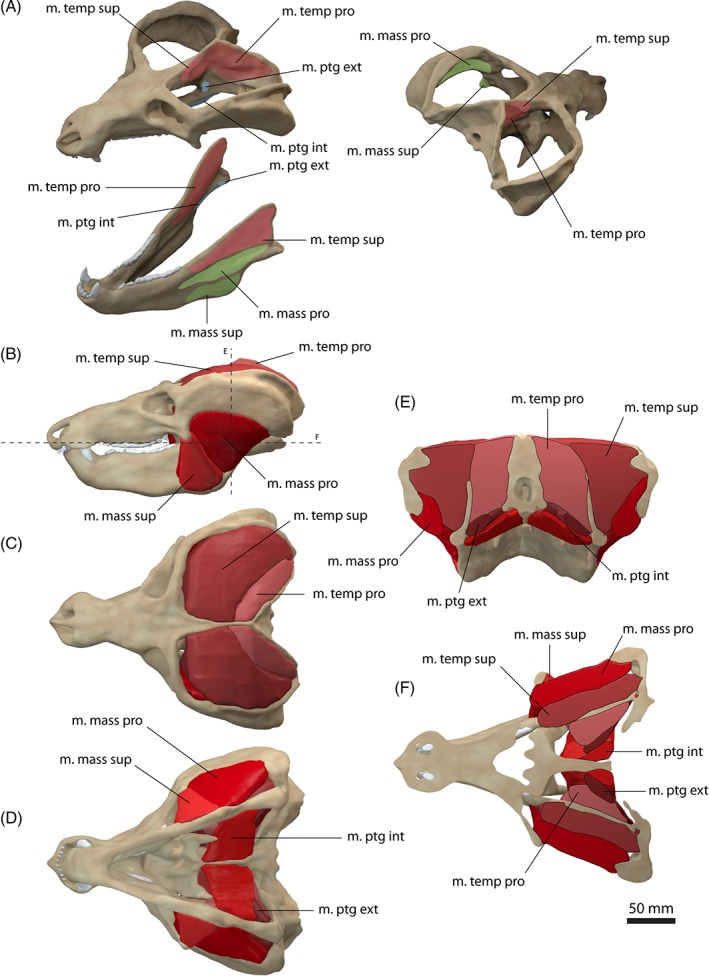
Jaw adductor musculature of *Diademodon tetragonus* *.* (A) Muscle origins and insertions on the skull and mandible. Muscle arrangement in (B) left lateral, (C) dorsal and (D) ventral view, (E) coronal and (F) horizontal section through the skull. See Section III.4 for muscle abbreviations.

*Diademodon tetragonus* represents a more derived stage of Cynodontia than *Thrinaxodon liorhinus* and this is partially reflected in the arrangement of the jaw adductor musculature. The adductor complex consists of the m. temporalis, the m. masseter and the m. pterygoideus, whereas the m. pseudotemporalis has most likely been lost (Fig. 7).

The m. temporalis is exceptionally large in *Diademodon tetragonus* due to the mediolateral expansion of the temporal region (Fig. 7B, C). The m. temporalis is likely subdivided into a superficial and a deep component as in *Thrinaxodon liorhinus*. The m. temporalis pars superficialis appears to have only a small origin anterior to the pars profunda (Figs 5C and 7A). The anterior extent of the attachment site is indicated by a slight widening of the postorbital. The m. temporalis pars superficialis covers the anterior portion of its deep counterpart and inserts onto the dorsal part of the lateral surface of the coronoid process. The insertion area is marked by a deeply excavated fossa (Brink, 1963) (Figs 5F and 7A). The ventral extent of the m. temporalis pars superficialis is indicated by a distinct ridge separating the fossa on the lateral surface of the coronoid approximately horizontally. The m. temporalis pars profunda occupies the posterior portion of the temporal region. It originates from the lateral surface of the parietal and anterior surface of the squamosal (Figs 5C and 7A). The attachment is demarcated by a prominent depression on both bones. The m. temporalis pars profunda inserts along the medial surface of the enlarged and anteroposteriorly expanded coronoid process of the dentary. The insertion extends anteriorly up to the level of the last tooth position, marked by a near‐vertical ridge (Figs 5F and 7A). Ventrally the muscle attachment is bounded by the suture to the post‐dentary bones.

In comparison to the condition found in *Thrinaxodon liorhinus* and similar to the m. temporalis, the m. masseter is probably substantially enlarged in *Diademodon tetragonus* (Fig. 7B, E). It is subdivided into a smaller superficial and a larger deep portion. The m. masseter pars superficialis originates from the conspicuous suborbital process of the jugal (Fig. 7A). The presence of a suborbital process has been cited as evidence for differentiation of the m. masseter (Allin & Hopson, 1992; Abdala & Damiani, 2004). The origin of the m. masseter pars superficialis extends onto the medial surface of the process. On the dentary, the m. masseter pars superficialis inserts onto the ventral part of the dentary below the fossa of the m. masseter pars profunda, from which its insertion area is separated by the lateral ridge of the dentary. The attachment is indicated by a shallow depression and slight medial angulation of the dentary (Figs 5F and 7A). The m. masseter pars profunda originates from the deeply excavated medial surface of the zygomatic arch. A prominent horizontal ridge of the squamosal indicates the dorsal extent of the muscle and appears to provide an increased surface area for muscle attachment suggesting the presence of a well‐developed m. masseter pars profunda (Fig. 7A, E). On the dentary, the muscle inserts in the depression between the m. temporalis pars superficialis and the m. masseter pars superficialis, marked by prominent ridges dorsally and ventrally (Figs 5F and 7A).

As with the other muscle groups, the m. pterygoideus is subdivided in *Diademodon tetragonus*. The m. pterygoideus externus originates from the anterolateral surface of the epipterygoid, marked by a shallow depression (Fig. 7A). The m. pterygoideus externus inserts into a small area anteromedial to the condylar process on the angular and the articular. The m. pterygoideus internus has its origin from the lateral and ventral surfaces of the pterygoid and the anterior portion of the basisphenoid process. The respective area is deeply notched suggesting a substantial attachment of the muscle (Fig. 7A). The m. pterygoideus internus likely attaches on the medial surface of the angular, although there are no clear osteological correlates visible on the bone. The insertion site is most likely restricted to the angular and does not extend onto the coronoid process, which is occupied by the m. temporalis profunda.

In contrast to *Thrinaxodon liorhinus*, there is no support for the presence of a m. pseudotemporalis in *Diademodon tetragonus*. The postorbital bar, from which the m. pseudotemporalis would originate, has been shifted anteriorly with respect to the zygomatic arch and the coronoid process of the dentary in *Diademodon tetragonus*. The course of the m. pseudotemporalis would therefore interfere with the eyeball and the m. temporalis. Similarly, the topology of the m. pterygoideus internus and the m. temporalis profunda would leave no space for the m. pseudotemporalis profundus. In addition, no osteological correlates (i.e. shallow depression on anteromedial surface of adductor chamber, sharp margins of postorbital bar, open mandibular adductor chamber) for either component of the m. pseudotemporalis are visible on the bone. Consequently, the m. pseudotemporalis was not reconstructed in *Diademodon tetragonus* and has most likely been lost at this stage of cynodontian evolution.

### (4) *Probelesodon sanjuanensis*

**Figure 8:**
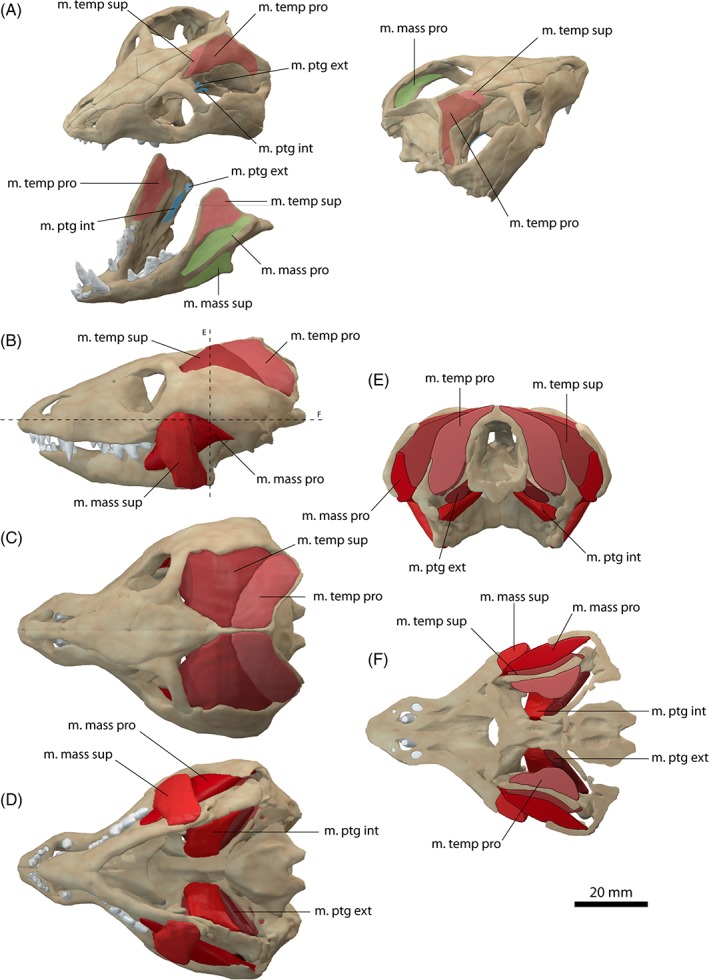
Jaw adductor musculature of *Probelesodon sanjuanensis* *.* (A) Muscle origins and insertions on the skull and mandible. Muscle arrangement in (B) left lateral, (C) dorsal and (D) ventral view, (E) coronal and (F) horizontal section through the skull. See Section III.4 for muscle abbreviations.

The arrangement of the jaw adductor musculature in *Probelesodon sanjuanensis* does not differ substantially from that of its precursors (see Sections IV.2 and IV.3). The adductor complex consists of the m. temporalis, the m. masseter and the m. pterygoideus, with each group subdivided into a superficial and a deep component (Fig. 8).

The m. temporalis pars superficialis arises from a small attachment anterior to the pars profunda. The origin appears to be restricted to the anterior portion of the parietal and is bounded by the suture with the postorbital, as indicated by the small size of the postorbital process and the expansion of the bone at this point (Fig. 8A). The m. temporalis pars superficialis overlies the pars profunda and increases in size anteroposteriorly towards its insertion on the lateral surface of the coronoid process (Fig. 8A, E). The attachment is restricted to the dorsal half of the latter marked by a shallow depression. A second depression for the attachment of the m. masseter pars profunda demarcates the ventral extent of the m. temporalis pars superficialis. The m. temporalis pars profunda forms the bulk of the m. temporalis group (Fig. 8E). It originates from the lateral surface of the parietal and the anterior surface of the squamosal, indicated by a shallow depression on the bone (Fig. 8A). The muscle inserts onto the medial surface of the coronoid process on the dentary. The insertion is demarcated by a shallow depression. A prominent horizontal ridge located at the level of the posteriormost tooth position marks the ventral extent of the attachment of the m. temporalis pars profunda.

In *Probelesodon sanjuanensis* the zygomatic arch is highly curved (Martinez & Forster, 1996) and very deep relative to other taxa studied here, suggesting a strong attachment of the m. masseter. The m. masseter pars superficialis originates from a deep depression on the ventral surface of the jugal just posterior to the suture with the maxilla (Fig. 8B, F). The extent of the attachment is well defined and continuous posteriorly up to the level of the postorbital contact. On the dentary, the m. masseter pars superficialis inserts onto the ventral part of the lateral surface marked by slight medial inflection and a shallow depression (Fig. 8A). The lateral ridge of the dentary marks the dorsal extent and the boundary with the m. masseter pars profunda. The latter originates from the medial surface of the zygomatic arch. As in *Diademodon tetragonus*, the bone surface is deeply excavated, suggesting a strong attachment of the muscle (Fig. 8E). The m. masseter pars profunda inserts into the fossa on the lateral surface of the dentary between the attachments of the m. temporalis pars superficialis and the m. masseter pars profunda. Two diagonal ridges mark the extent of the insertion. Anteriorly, the m. masseter pars profunda extends to the level of the posterior tooth position.

The m. pterygoideus is most likely subdivided into an internal and an external part (Fig. 8D). The m. pterygoideus externus originates from the anteroventral surface of the epipterygoid, marked by a distinct depression on the lateral surface of the bone. It inserts onto the medial surface of the articular, marked by a small circular depression (Fig. 8A). The m. pterygoideus internus originates from the ventrolateral margin of the pterygoid. Compared to *Thrinaxodon liorhinus* and *Diademodon tetragonus*, the attachment is not marked by a sharp ridge or notch, but by a shallow depression between the pterygoid flange and the alisphenoid. The m. pterygoideus internus appears to insert onto the angular and prearticular, as indicated by a shallow depression, but the attachment is not very clear (Fig. 8A). Alternatively, the attachment might be located on the dentary, below the insertion of the m. temporalis profunda.

As in *Diademodon tetragonus*, there is no evidence that a m. pseudotemporalis group might have been present in *Probelesodon sanjuanensis*. The anterior position of the postorbital bar would result in a conflict of the m. pseudotemporalis superficialis with the m. temporalis. Similarly, the reduction of the post‐dentary bones and the closing of the mandibular adductor fossa leave no space for the insertion on the mandible.

### (5) *Probainognathus*

**Figure 9:**
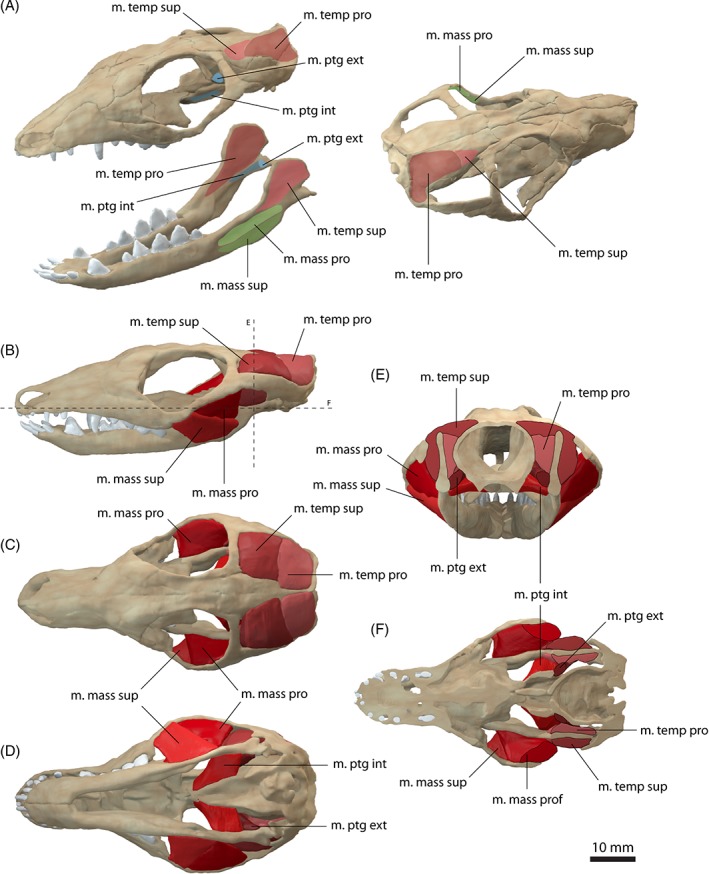
Jaw adductor musculature of *Probainognathus* sp. (A) Muscle origins and insertions on the skull and mandible. Muscle arrangement in (B) left lateral, (C) dorsal and (D) ventral view, (E) coronal and (F) horizontal section through the skull. See Section III.4 for muscle abbreviations.

*Probainognathus* exhibits the mammal‐like muscle division of the m. temporalis, the m. masseter and the m. pterygoideus (Fig. 9). The m. temporalis pars superficialis originates from the anterolateral surface of the parietal. The attachment is marked by a faint depression and bounded anteriorly by the postorbital bar and posteriorly by the attachment of the m. temporalis pars profunda (Fig. 9A). The m. temporalis pars superficialis inserts onto the lateral surface of the coronoid process of the dentary, indicated by a shallow depression. Ventrally, the extent of the muscle is marked by the lateral ridge of the dentary confluent with the surangular (Fig. 9A). The insertion extends to the base of the coronoid process anteriorly. The m. temporalis profunda is partially covered by the pars superficialis and originates posteriorly to the latter (Fig. 9A, E). The attachment of the pars profunda is indicated by a distinct depression on the lateral surface of the parietal and along the sagittal and occipital crests. A vertical ridge on the parietal separates the attachment sites of the temporalis muscle bodies. The pars profunda inserts on the medial surface of the coronoid process. The insertion extends ventrally to the contact with the postdentary bones and anteriorly to the base of the coronoid process (Fig. 9A). In comparison to other non‐mammalian cynodonts, the temporalis muscle group is not as prominently developed in the studied specimen of *Probainognathus*. This is due to the fact that the temporal region and the width of the zygomatic arch are not notably expanded considering the immature stage of the specimen. However, it can be assumed that fully mature individuals, which show the typically expanded temporal morphology (Romer, 1970), would be more comparable to other taxa, such as *Probelesodon* in the morphology of the m. temporalis.

The m. masseter pars superficialis arises from the ventromedial surface of the jugal/maxilla contact (Fig. 9A, B, E). The origin is clearly indicated by the characteristic suborbital angulation of the zygomatic arch (Abdala & Damiani, 2004). The muscle inserts along a dorsoventrally short, but anteroposteriorly elongate region on the ventrolateral surface of the dentary. The m. masseter pars profunda originates from the medial surface of the jugal (Fig. 9A, E). The attachment extends posteriorly to the level of the postorbital process. A further posterior extent of the attachment is unlikely as the muscle would interfere with the m. temporalis superficialis. On the dentary, the m. masseter pars profunda inserts into a weakly indicated fossa. Its extent is comparable to that of the pars superficialis. The juvenile specimen of *Probainognathus* is missing a distinct angle and expanded posteroventral region of the dentary found in adult stages (Romer, 1970). In fully grown individuals, the insertion of the m. masseter was most likely more substantial and would occupy a proportionally larger area.

The m. pterygoideus externus was reconstructed to originate from the ventrolateral surface of the epipterygoid (Fig. 9A, D, E). The respective area is only partially preserved, but the origin of the m. pterygoideus in more basal as well as in more derived taxa from the epipterygoid/alisphenoid region strongly suggests a similar attachment. The m. pterygoideus externus inserts onto a small depression on the medial surface of the articular anteromedial to the condylar process (Fig. 9A). The m. pterygoideus internus originates from the ventrolateral surface of the posterior region of the pterygoid adjacent to the pterygoid flange. It is unclear whether the attachment extended onto the latter, but the smooth surface of the pterygoid flange and the lateral orientation makes an attachment unlikely. The m. pterygoideus internus inserts along the postdentary bones (prearticular, angular) along an anteroposteriorly elongate attachment ventral to the m. temporalis pars profunda (Fig. 9A, D). In adult individuals of *Probainognathus,* which exhibit a prominent dentary angle, an additional/extended insertion of the m. pterygoideus internus is possible. The ventromedial surface of the dentary would form a prominent attachment for the m. pterygoideus not present in the immature specimen. Thus, in adult individuals an extension of the pterygoideus internus onto the dentary might have occurred in *Probainognathus*.

### (6) *Morganucodon oehleri*

**Figure 10:**
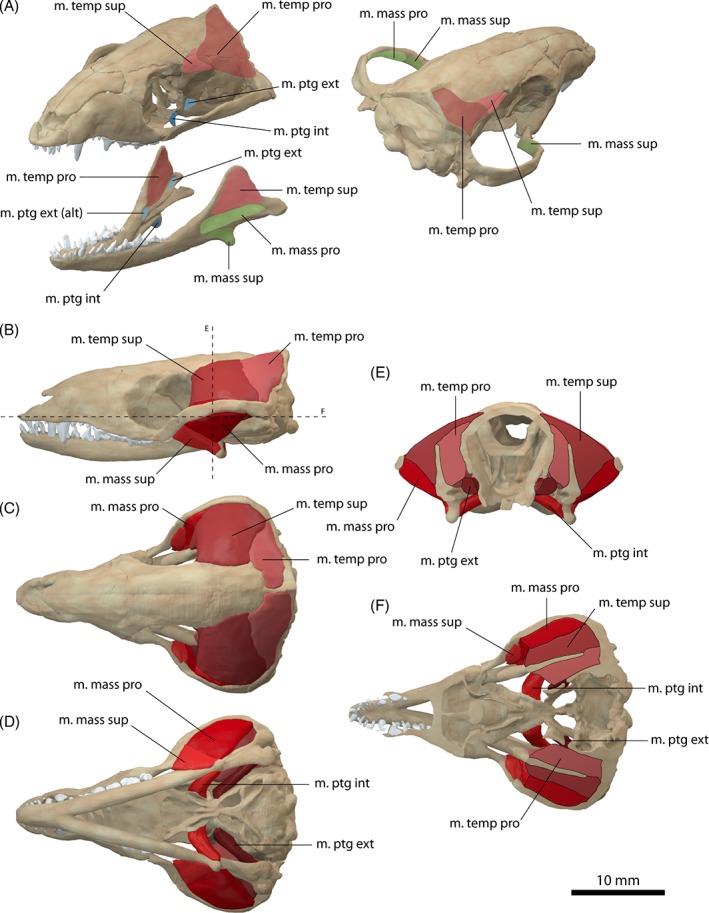
Jaw adductor musculature of *Morganucodon oehleri* *.* (A) Muscle origins and insertions on the skull and mandible; (alt) indicates alternate muscle attachment as discussed in text. Muscle arrangement in (B) left lateral, (C) dorsal and (D) ventral view, (E) coronal and (F) horizontal section through the skull. See Section III.4 for muscle abbreviations.

The jaw adductor musculature of the basal mammaliaform *Morganucodon oehleri* is generally similar to that of the aforementioned taxa regarding the subdivision of the individual muscle groups. However, the arrangement (location and attachment) of the specific muscles reflects the transitional state of the jaw joint modification and overall changes in the cranial morphology, such as the loss of the postorbital (Fig. 10). The m. temporalis is subdivided into a superficial and deep portion. The m. temporalis pars superficialis originates from a relatively small region on the anterolateral surface of the parietal and the alisphenoid (Fig. 10A). The attachment is only weakly demarcated. As the postorbital has been lost in *Morganucodon oehleri* there is no clear boundary for the anterior extent of the muscle attachment. The frontal is slightly expanded mediolaterally relative to the parietal, indicating that the attachment of the m. temporalis pars superficialis is restricted to the parietal and bounded anteriorly by the frontal/parietal and the frontal/alisphenoid sutures. The m. temporalis pars superficialis inserts onto the dorsolateral surface of the coronoid process (Figs 5G and 10A). A faint horizontal ridge just above the level of the condyle marks the separation between the insertions of the m. temporalis pars superficialis and the m. masseter pars profunda (Figs 5G and 10A). Kermack *et al*. (1973) indicated the attachment of the temporalis muscle on the lateral surface of the coronoid process. The m. temporalis pars profunda has an extensive origin from the dorsolateral surface of the parietal, the dorsal portion of the petrosal and the anterior surface of the squamosal (Figs 5D and 10A). The attachment extends onto the sagittal and occipital crests. The m. temporalis pars profunda inserts on the medial surface of the coronoid process (Figs 5H and 10A). The attachment is bounded ventrally by a prominent horizontal ridge (‘medial flange’, Kermack *et al*., 1973). Kermack *et al*. (1973) indicated this region as attachment of the pterygoideus externus muscle and did not recognise a subdivision of the m. temporalis (although the muscle is referred to as ‘deep temporal muscle’). In comparison with other cynodont and extant taxa, the attachment of the m. pterygoideus externus onto the coronoid process would be unique in *Morganucodon oehleri* and thus be very unlikely.

The m. masseter pars superficialis originates from the medial surface of the anterior portion of the jugal (Fig. 10A). Unlike in non‐mammaliaform cynodonts the attachment does not extend to the maxilla/jugal contact due to a distinct kink in the jugal. An attachment anterior to it is unlikely as there is only limited space between the jugal and the pterygoid. The m. masseter pars superficialis might extend at least partially onto the zygomatic arch as found in extant mammals, but the exact attachment cannot be identified with certainty, as large portions of the zygomatic arch had to be reconstructed (see online Figure S5). On the dentary, the m. masseter pars superficialis inserts into a prominent, triangular depression dorsal to the dentary angle (Figs 5G and 10A). The attachment is demarcated dorsally by a horizontal ridge confluent with the condylar process. Kermack *et al*. (1973) identified this region as the attachment of the masseter muscle, but did not recognise a subdivision of the m. masseter. The m. masseter pars profunda most likely originates from the medial surface of the zygomatic arch, but the lack of preserved fossil elements prohibits an exact identification of the attachment. The size of the mandibular attachment suggests that the origin extended the whole length of the zygomatic arch and onto the squamosal. The m. masseter pars profunda inserts on the fossa on the lateral surface of the coronoid process ventral to the attachment of the m. temporalis pars superficialis. The attachment is marked by a shallow, elongate depression, which extends from the condylar process to the base of the coronoid process anteriorly. A separate origin and insertion of the m. masseter pars profunda were not identified by Kermack *et al*. (1973).

The m. pterygoideus externus originates from the ventral part of the lateral surface of the alisphenoid (Fig. 10A). The attachment is clearly circumscribed by a circular depression bordered by slightly raised and thickened anterior and dorsal ridges. The insertion on the mandible of the m. pterygoideus externus is ambiguous. Following the pattern of non‐mammaliaform cynodonts, the muscle would insert on the medial surface of the condylar region anterior to the dentary condyle. Kermack *et al*. (1973) suggested an attachment of the m. pterygoideus externus on the thickened region around the coronoid boss. The lack of osteological correlates makes a definite identification of the mandibular attachment difficult. However, the attachment of the m. pterygoideus externus to the medial surface of the condylar process in *Monodelphis domestica* and the analogous position of the attachment lateral to the jaw joint tentatively indicates the attachment near the condylar process. The m. pterygoideus internus originates from the ventrolateral surface of the pterygoid and the pterygoid flange as indicated by a lateral ridge (Fig. 10A). The prominent development of the dentary angle suggests an insertion of the muscle on its medial surface (Fig. 5H) rather than the considerably reduced postdentary bones, comparable to the condition discussed for *Probainognathus*.

### (7) *Hadrocodium wui*

**Figure 11:**
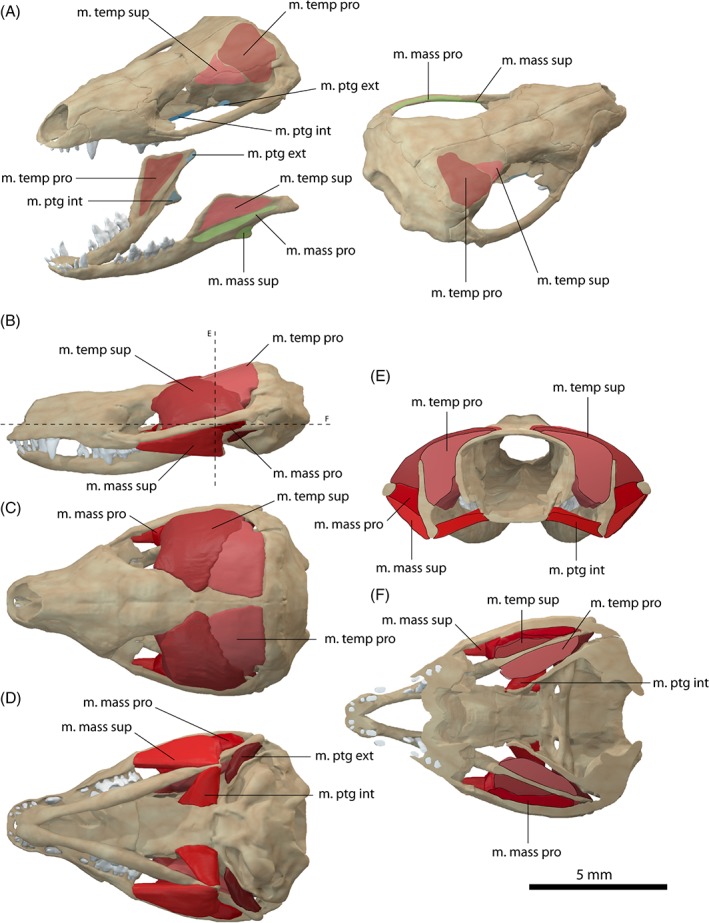
Jaw adductor musculature of *Hadrocodium wui* *.* (A) Muscle origins and insertions on the skull and mandible. Muscle arrangement in (B) left lateral, (C) dorsal and (D) ventral view, (E) coronal and (F) horizontal section through the skull. See Section III.4 for muscle abbreviations.

The division and arrangement of the jaw adductor complex (Fig. 11) in *Hadrocodium wui* is comparable to that of *Morganucodon oehleri*. *Hadrocodium wui* appears to be one of the first mammaliaforms to have achieved the full mammalian condition. The m. temporalis pars superficialis originates from the anterolateral surface of the parietal and the dorsolateral surfaces of the alisphenoid and the petrosal (Fig. 11A). Similar to the condition in *Morganucodon oehleri* the frontal expands in mediolateral width indicating the anterior extent of the m. temporalis pars superficialis. The ventral extent of the muscle is marked by a horizontal swelling on the lateral surface of the alisphenoid and the petrosal in *Hadrocodium wui*. The m. temporalis pars superficialis inserts on the lateral surface of the coronoid process (Fig. 11A). The attachment is marked by a prominent depression. The ventral extent is bounded by a faint ridge marking the boundary to the m. masseter pars profunda (Fig. 11A, E). The m. temporalis pars profunda originates from the dorsolateral surface of the parietal and the lateral surface of the petrosal (Fig. 11A, E). The attachment covers the region lateral and anterior to the sagittal mideline of the skull roof and the weakly developed occipital crest. Unlike in other cynodonts and mammaliaforms, the m. temporalis pars profunda appears not to arise from the squamosal in *Hadrocodium wui*. The dorsal margin of the squamosal tapers to a thin margin, which offers no attachment surface for the muscle. The m. temporalis pars profunda inserts on the medial surface of the coronoid process (Fig. 11A). As in *Morganucodon oehleri* the ventral extent is demarcated by a prominent horizontal ridge located at the level of the dentary condyle.

**Figure 12:**
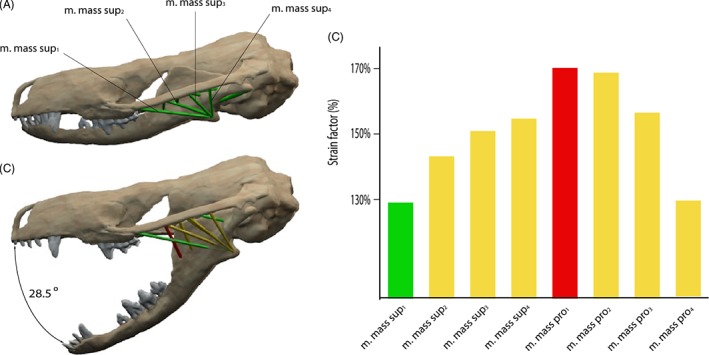
Strain analysis for different muscle positions of the m. masseter pars superficialis in *Hadrocodium wui*. Mandible in closed (A) and maximum gape (B) position with different muscle arrangements of the m. masseter pars superficialis (m. mass sup_1–4_). (C) Muscle strain ratios for different muscle arrangements. Colour coding indicates strain ratios below 130% (green), between 130 and 170% (yellow) and over 170% (red). See Section III.4 for muscle abbreviations.

The m. masseter pars superficialis originates from the anterior portion of the jugal. Anteriorly, the attachment extends to the contact with the maxilla (Fig. 11A). Similar to *Morganucodon oehleri*, a large part of the zygomatic arch is not preserved in *Hadrocodium wui* making exact identification of the muscle attachment difficult. Strain analyses of different muscle positions along the zygomatic arch indicate that the m. masseter pars superficialis could have extended to the anterior half of the zygomatic arch. As shown in Fig. 12, muscle fibres attaching to the anterior half of the zygomatic arch would allow a relatively large gape angle (28.5°), without reaching the maximum muscle tension limit of 170%. Muscle fibres originating further posteriorly, as well as the m. masseter pars profunda considerably restrict the gape angle. The m. masseter pars superficialis inserts on the prominently developed dentary angle. The insertion is demarcated by a small triangular depression. The m. masseter pars profunda originates from the medial surface of the zygomatic arch. Although the zygomatic arch is not preserved, the muscle attachment is well constrained by the location of the adjacent muscles (Fig. 11). The m. masseter pars profunda inserts along a dorsoventrally narrow but anteroposteriorly elongate depression on the lateral surface of the dentary.

The m. pterygoideus externus arises from the ventrolateral surface of the petrosal (Fig. 11A). The attachment is marked by a small depression on the ventral portion of the bone. The muscle inserts on the medial surface of the condylar process (Fig. 11A, D). As in *Morganucodon oehleri*, the insertion is not well constrained. *Hadrocodium wui* lacks a thickened coronoid boss, which could serve as an alternative attachment as suggested for *Morganucodon oehleri* (see Section IV.6). The muscle was therefore reconstructed to attach adjacent to the condyle as in *Monodelphis domestica*. The m. pterygoideus internus originates from the ventrolateral surface of the pterygoid, although this region is incompletely preserved and missing the pterygoid flange (Fig. 11A, E). However, the m. pterygoideus internus consistently arises from this region in other mammaliaforms and cynodonts and a lack of alternative origins makes this the most likely location. The muscle inserts on the medial surface of the dentary angle.

## V. EVOLUTIONARY TRENDS

The complexity of morphological transformations occurring throughout the evolutionary history of mammals makes it difficult to disentangle individual evolutionary trends and specific contributions of different hard‐ and soft‐tissue structures. Transformations of osteological structures are comparably well documented in the fossil record and have consequently been discussed in great detail (e.g. Crompton, 1963; Allin, 1975; Sidor, 2001, 2003; Luo, 2007, 2011). While this is more difficult for soft tissues, three‐dimensional reconstructions can permit the identification of evolutionary trends (e.g. Rowe *et al*., 2011), including the modification of the jaw adductor musculature as presented here.

Several trends become obvious when comparing musculoskeletal arrangements across different cynodont and mammaliaform taxa. Chief among these trends is the separation and subdivision of the individual muscle groups. While there is little doubt that the temporalis musculature had completely subdivided in basal cynodonts due to the elevation and posterior displacement of the coronoid process and a concomitant transfer of the muscle to the dentary (Barghusen, 1968, 1972; Bramble, 1978; Abdala & Damiani, 2004), the timing of the subdivision of the masseter muscle has been contentious. A number of authors argued for an undivided masseter muscle arising from the medial surface of the jugal and the zygomatic arch, respectively (Barghusen, 1968, 1972, 1973; Demar & Barghusen, 1972; Crompton & Parker, 1978), citing the lack of osteological features for separate muscle origins as evidence. Accordingly, these authors suggested the emergence of a fully subdivided masseter in *Trirachodon*, *Probainognathus* and more‐derived eucynodonts. However, osteological correlates for a separate cranial origin of a subdivided masseter muscle have been suggested in the form of a suborbital angulation (as in *Thrinaxodon liorhinus*) and suborbital process (as in *Diademodon tetragonus*) of the jugal (Allin & Hopson, 1992; Abdala & Damiani, 2004) occurring in Epicynodontia. Distinctly separated mandibular insertions for a superficial and a deep masseter component in *Thrinaxodon liorhinus* (see Section IV.2; Fig. 4) would lend further support for masseter subdivision to have been completed in basal epicynodonts.

**Figure 13:**
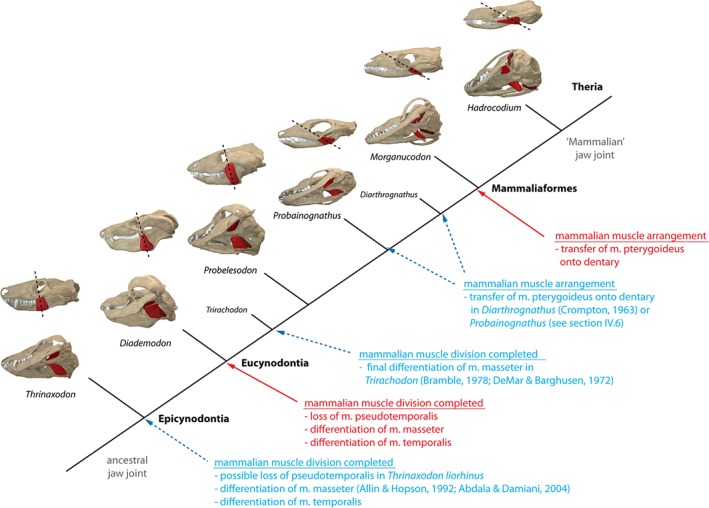
Evolutionary trends in musculoskeletal arrangement across the cynodont–mammal transition. Orientation of m. masseter pars superficialis is shown in the top row of models, with position of the m. pterygoideus group shown below. Red arrows indicate evolutionary events based on this study; blue arrows indicate alternative hypotheses for transformational events.

Assuming the masseter muscle had subdivided in *Thrinaxodon liorhinus*, a successive reorientation of the m. masseter pars superficialis can be observed throughout the cynodont–mammalian transition (Fig. 13). As reconstructed here, the m. masseter pars superficialis shows a nearly vertical orientation in *Thrinaxodon liorhinus* and *Diademodon tetragonus* and the same has been noted for other eucynodont taxa, such as *Trirachodon* (Reed *et al*., 2016). First seen here in *Probelesodon sanjuanensis,* and more distinctly in *Probainognathus*, the m. masseter pars superficialis shifts into a more inclined position due to the posterior expansion of the dentary in these taxa. With the development of a distinct dentary angle and further posterior displacement in *Morganucodon oehleri* and more derived taxa, the m. masseter pars superficialis reaches an anteroposteriorly diagonal orientation of approximately 45° to the horizontal.

Among the different jaw adductors, the pterygoideus muscles are subject to the highest degree of interpretation reflecting the uncertainties regarding the individual attachments and paucity of osteological correlates. Debate largely centres on the mandibular attachment, whether the individual muscle subdivision inserted on the postdentary bones or the dentary, and at which stage a shift from the one to the other might have occurred. A dentary insertion for the pterygoideus muscles was suggested for a number of taxa, such as *Thrinaxodon liorhinus* and *Trirachodon* (Crompton, 1963; Kemp, 1972*a*), with the m. pterygoideus internus inserting on the medial surface of the dentary angle. Alternatively, the attachment of the pterygoideus muscles on the postdentary bones, in particular the medial surface of the angular, has been proposed for basal cynodonts, including *Thrinaxodon liorhinus* (Watson, 1953; Barghusen, 1968; Allin, 1975). The possibility that the muscle scars on the medial surface of the dentary angle could result from the attachment of the digastric and not the pterygoideus musculature tentatively supports the latter assumption. In consideration of the size of the postdentary bones and the presence of (admittedly faint) depressions indicating attachment on the angular, the pterygoideus musculature was reconstructed here inserting on the postdentary elements in *Thrinaxodon liorhinus* and *Diademodon tetragonus*. The first indication of a shift of the pterygoideus musculature is provided by the well‐developed dentary angle in *Morganucodon oehleri*, which most likely served as the attachment for the m. pterygoideus internus. This modification of the muscle insertion, however, could have occurred already in *Diarthrognathus broomi*, considering the posterior expansion of the dentary angle and the concomitant reduction of postdentary bones (Crompton, 1963). This would shift the transfer of at least parts of the pterygoideus musculature (i.e. m. pterygoideus internus) to below Mammaliaformes (Fig. 13). It is noteworthy that different ontogenetic stages of *Probainognathus* exhibit morphological variations in the expression of the dentary angle and therefore of the potential mandibular insertions of the m. pterygoideus internus (see Section IV.5). Although the weakly developed dentary angle in the juvenile individual studied here suggests that the m. pterygoideus internus was restricted to the postdentary bones, adult specimens exhibit a well‐developed dentary angle. Consequently, the muscular attachment might have extended (although not fully shifted) with ontogeny. It has been shown that osteological correlates for muscle attachments can be subject to ontogeny in *Thrinaxodon liorhinus* and allometric trends can be very variable for different muscle groups (Jasinoski *et al*., 2015). Osteological correlates relating to the attachment of masseter musculature were found to increase with negative allometry (relatively less prominent), whereas those relating to the temporalis muscles were positively allometric (relatively more prominent). While the lack of a range of specimens precludes clear evidence for comparable ontogenetic variations in *Probainognathus*, this shows that care should be taken when considering osteological correlates. Further caution is warranted due to the unstable taxonomic status of the specimen of *Probainognathus* used in this study. Future phylogenetic analyses might be able to resolve this issue, allowing a more precise identification of the timing of musculoskeletal evolution.

**Figure 14:**
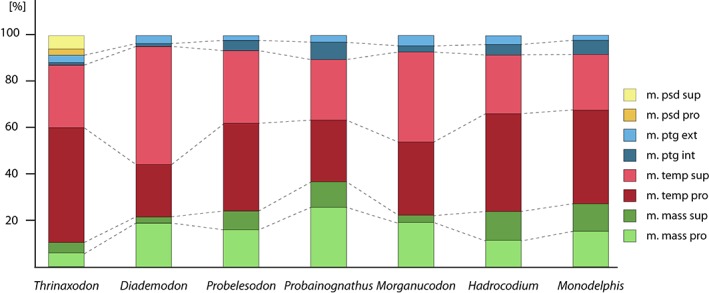
Relative muscle volumes for the individual jaw adductor muscles. See Section III.4 for muscle abbreviations.

Apart from the rearrangement of the jaw adductor musculature observed to occur in parallel with the decrease in size and presumed stability of the quadrate–articular jaw joint, an increase in muscle mass, and therefore muscle force, has been postulated (e.g. Watson, 1912; Adams, 1918; Crompton, 1963; Barghusen, 1973; Reed *et al*., 2016). However, based on the current reconstructions, relative muscle mass does not appear to show a clear trend of increasing towards derived cynodonts and mammaliaforms (Fig. 14). Notable differences only occur in the relative proportions of the temporalis muscle subdivisions in *Diademodon tetragonus*, showing an enlarged m. temporalis pars superficialis in relation to its deep counterpart. Although it cannot be ruled out that this reflects uncertainties in the reconstruction method, it seems unlikely as potential errors in muscle reconstruction are intrinsic to the reconstruction of all taxa. It is possible that this pattern reflects a case of dietary specialisation in *Diademodon tetragonus*, for which omnivorous or herbivorous adaptations have been suggested (Grine, 1977; Botha *et al*., 2005). As reconstructed, *Morganucodon oehleri* shows similar relative muscle proportions. However, *Morganucodon* is considered to be a dietary specialist (adapted to a high proportion of hard‐shelled insects within its diet) (Gill *et al*., 2014), which would suggest that relative muscle proportions might contain a dietary signal.

## VI. LIMITATIONS AND SCOPE FOR FUTURE DIRECTIONS

Due to the nature of fossils, soft‐tissue reconstructions and their accuracy depend to a considerable degree on the quantity and quality of preserved specimens. Distinguishing between taphonomic artefacts, pathological malformations and the actual *in vivo* condition is a major challenge, not just for digital restoration and reconstruction of fossils, but for palaeontological studies in general. It is therefore sensible to compare different specimens of the same taxon and this approach has been taken here where possible. However, given that some species in this study are represented by a single fossil specimen, information provided by these fossils might be incomplete, prone to intraspecific variation or variation with ontogenetic stage (see comments on *Probainognathus*, Section III.1*d*).

The method applied here for the reconstruction of the jaw adductor musculature further relies on constraints imposed by the osteological structure. In comparison to other vertebrate groups, such as archosaurs, in which the cranial bones encase the musculature, cynodont and mammaliaform skulls are relatively open. Exact muscle boundaries are therefore sometimes difficult to establish, which could influence the volumes of the digital muscle reconstructions. However, by tying in multiple lines of evidence from comparisons with extant taxa, a phylogenetically grounded framework and additional analyses (including strain calculations), uncertainties can be minimised. Still, it is important to note that the reconstructions presented and discussed here do not represent an endpoint but the current summary of knowledge and a working hypothesis. These may be modified in the light of new fossil discoveries and new technological advances and methods.

Despite some uncertainties, the digital and three‐dimensional nature of the osteological and muscle reconstructions can pave the way for further analysis. Computational analyses, such as finite element analysis (FEA) (Rayfield, 2007) and multibody dynamics analysis (MDA) (Curtis *et al*., 2010; Lautenschlager *et al*., 2016), hold the potential to test biomechanical and functional aspects of mammalian jaw adductor evolution. Using the information presented and reviewed herein as a stepping stone will permit future research to test competing hypothesis regarding muscle arrangement. We envision that such analyses could be used, for example, to clarify aspects of the attachment and orientation of the m. masseter pars superficialis or the shift of the pterygoideus musculature onto the dentary and the resulting functional consequences. A number of studies on the functional implications relating to the anatomical transformations taking place in the skull and lower jaw across the cynodont–mammal transition have been conducted, using simplified or theoretical two‐ and three‐dimensional models (Demar & Barghusen, 1972; Greaves, 1974, 1991; Bramble, 1978; Reed *et al*., 2016). Modern computational analysis techniques represent a logical progression building on previous hypotheses and theoretical assumptions. The jaw adductor muscles reconstructed here are primarily responsible for jaw movement in the vertical plane (dorsoventral motion), as they occur in morganucodontids and extant didelphids. Theoretical calculations based on these reconstructions, such as lever mechanics (Lautenschlager, 2013), would largely neglect palinal components of jaw motion and cranial kinesis (where present), which were likely present in tritylodontids, haramiyidans and multituberculates (Meng *et al*., 2014; Luo *et al*., 2015). However, these more‐complex properties may be incorporated and analysed in kinematic models using MDA and FEA.

In addition, data from extant taxa and developmental studies hold further potential to elucidate patterns of musculoskeletal development (Anthwal, Joshi & Tucker, 2013; Ramirez‐Chaves *et al*., 2016). Coupled with new imaging techniques, data from fossil taxa and biomechanical analyses, investigation of the evolution of soft tissues from different perspectives is likely to be the most productive and successful.

## VII. CONCLUSIONS

(1) The origin of mammals from non‐mammalian synapsid ancestors is a key event in vertebrate evolutionary history and a classic, textbook example of an evolutionary transition. Concomitant to unlocking the sequence of hard‐tissue transformations, numerous studies have focussed on the arrangement and role of the jaw adductor musculature. While central to the debate on feeding and jaw joint–middle ear evolution, most published muscle reconstructions have largely been restricted to two‐dimensional schematics and remained unclear in the exact extent and location of muscles.

(2) Taking advantage of recently documented fossils that represent new specimens of existing species with data derived from CT scanning and a suite of novel digital restoration, reconstruction and modelling techniques, a revised perspective on the morphology and arrangement of the jaw adductor system is presented and discussed.

(3) Current evidence suggests that the mammal‐like division of the jaw adductor musculature (into deep and superficial components of the m. masseter, the m. temporalis and the m. pterygoideus) was completed in Eucynodontia. The m. pseudotemporalis muscle group, plesiomorphic for synapsids, was likely retained in the epicynodont *Thrinaxodon liorhinus*, but successively lost in more‐derived taxa.

(4) The arrangement of the jaw adductor musculature in a mammalian fashion, with the m. pterygoideus group inserting on the dentary, was completed in basal Mammaliaformes as suggested by the muscle reconstruction of *Morganucodon oehleri*. A partial transfer of the pterygoideus musculature onto the dentary most likely occurred in taxa basal to Mammaliaformes such as *Diarthrognathus broomi* or possibly *Probainognathus*.

(5) Consequently, transformation of the jaw adductor musculature from the ancestral (‘reptilian’) to the mammalian condition must have preceded the emergence of Mammalia and the full formation of the mammalian jaw joint and the definitive mammalian middle ear. This suggests that the modification of the jaw adductor system played a pivotal role in the functional morphology and biomechanical stability of the jaw joint.

(6) Technological advances in the form of novel computational analyses techniques, such as finite element analysis and multibody dynamics analysis, offer exciting new research possibilities, allowing the testing of different hypotheses and the opportunity to clarify current uncertainties. Importantly, research on extant taxa and in the field of developmental biology will help to shed more light on anatomical details from a different perspective.

## Supporting information


**Figure S1.** Restored osteology of *Thrinaxodon liorhinus*.

**Figure S2.** Restored osteology of *Diademodon tetragonus*.

**Figure S3.** Restored osteology of *Probelesodon sanjuanensis*.

**Figure S4.** Restored osteology of *Probainognathus* sp.

**Figure S5.** Restored osteology of *Morganucodon oehleri*.

**Figure S6.** Restored osteology of *Hadrocodium wui*.

**Figure S7.** Interactive 3D PDF of *Thrinaxodon liorhinus* containing the digital model of the restored osteology and the reconstructed musculature.

**Figure S8.** Interactive 3D PDF of *Diademodon tetragonus* containing the digital model of the restored osteology and the reconstructed musculature.

**Figure S9.** Interactive 3D PDF of *Probelesodon sanjuanensis* containing the digital model of the restored osteology and the reconstructed musculature.

**Figure S10.** Interactive 3D PDF of *Probainognathus* sp. containing the digital model of the restored osteology and the reconstructed musculature.

**Figure S11.** Interactive 3D PDF of *Morganucodon oehleri* containing the digital model of the restored osteology and the reconstructed musculature.

**Figure S12.** Interactive 3D PDF of *Hadrocodium wui* containing the digital model of the restored osteology and the reconstructed musculature.

**Figure S13.** Interactive 3D PDF of *Monodelphis domestica* containing the digital model of the restored osteology and the reconstructed musculature.
