# Supplementary material for: Morphological evolution of the mammalian jaw adductor complex
Source: Biol Rev Camb Philos Soc. 2016 Nov 23;92(4):1910–40. doi: 10.1111/brv.12314 (PMC6849872; doi:10.1111/brv.12314)
Supplement: Supplementary file 1 — Figure S1. Restored osteology of Thrinaxodon liorhinus. [file BRV-92-1910-s001.pdf]

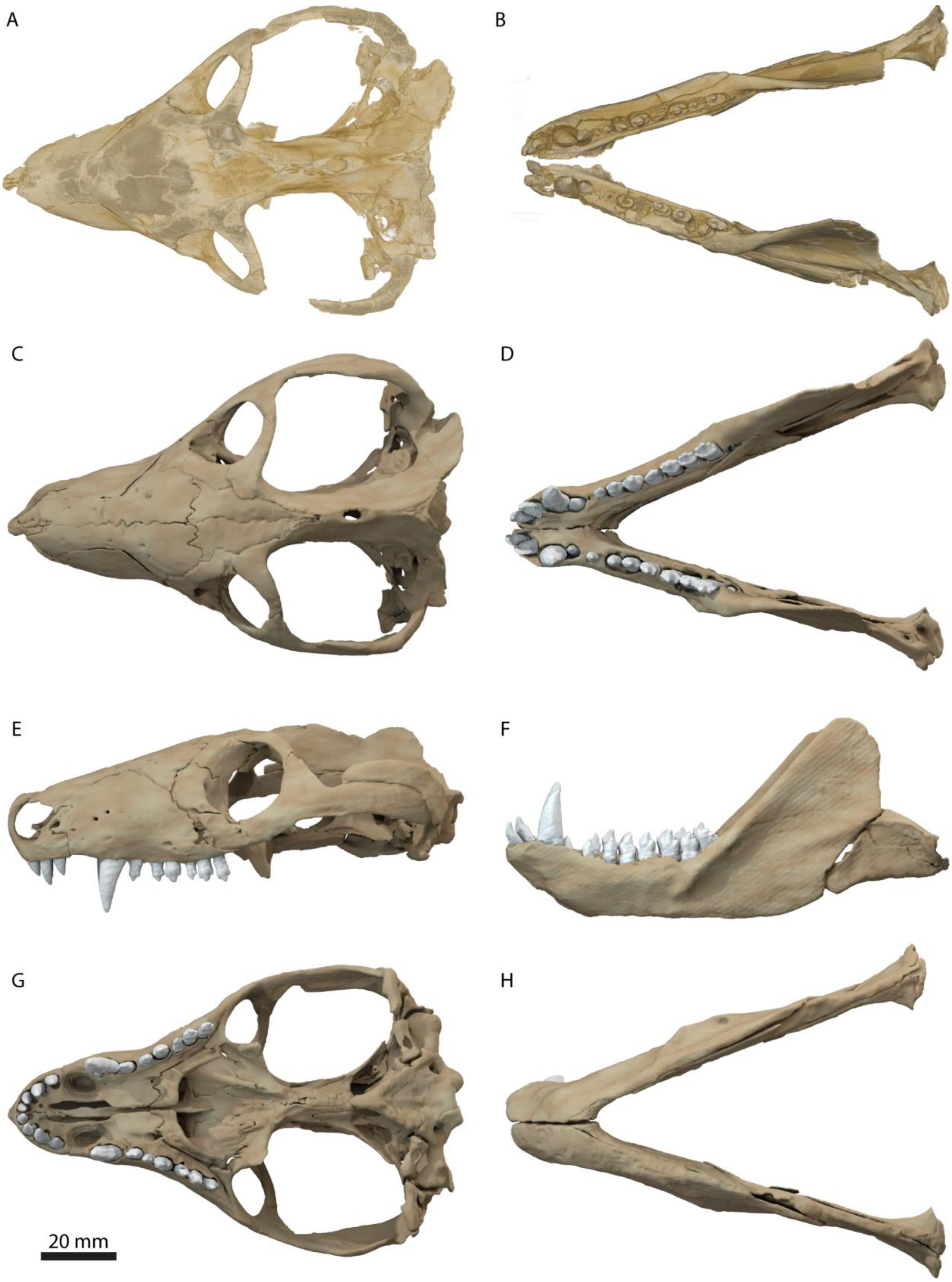

Fig. S1. Restored osteology of *Thrinaxodon liorhinus*. Digital models of the original (A) and restored (C E, G) skull and the original (B) and restored (D, F, H) lower jaw in (A–D) dorsal, (E, F) left lateral and (G, H) ventral views.
