# Supplementary material for: Morphological evolution of the mammalian jaw adductor complex
Source: Biol Rev Camb Philos Soc. 2016 Nov 23;92(4):1910–40. doi: 10.1111/brv.12314 (PMC6849872; doi:10.1111/brv.12314)
Supplement: Supplementary file 2 — Figure S2. Restored osteology of Diademodon tetragonus. [file BRV-92-1910-s002.pdf]

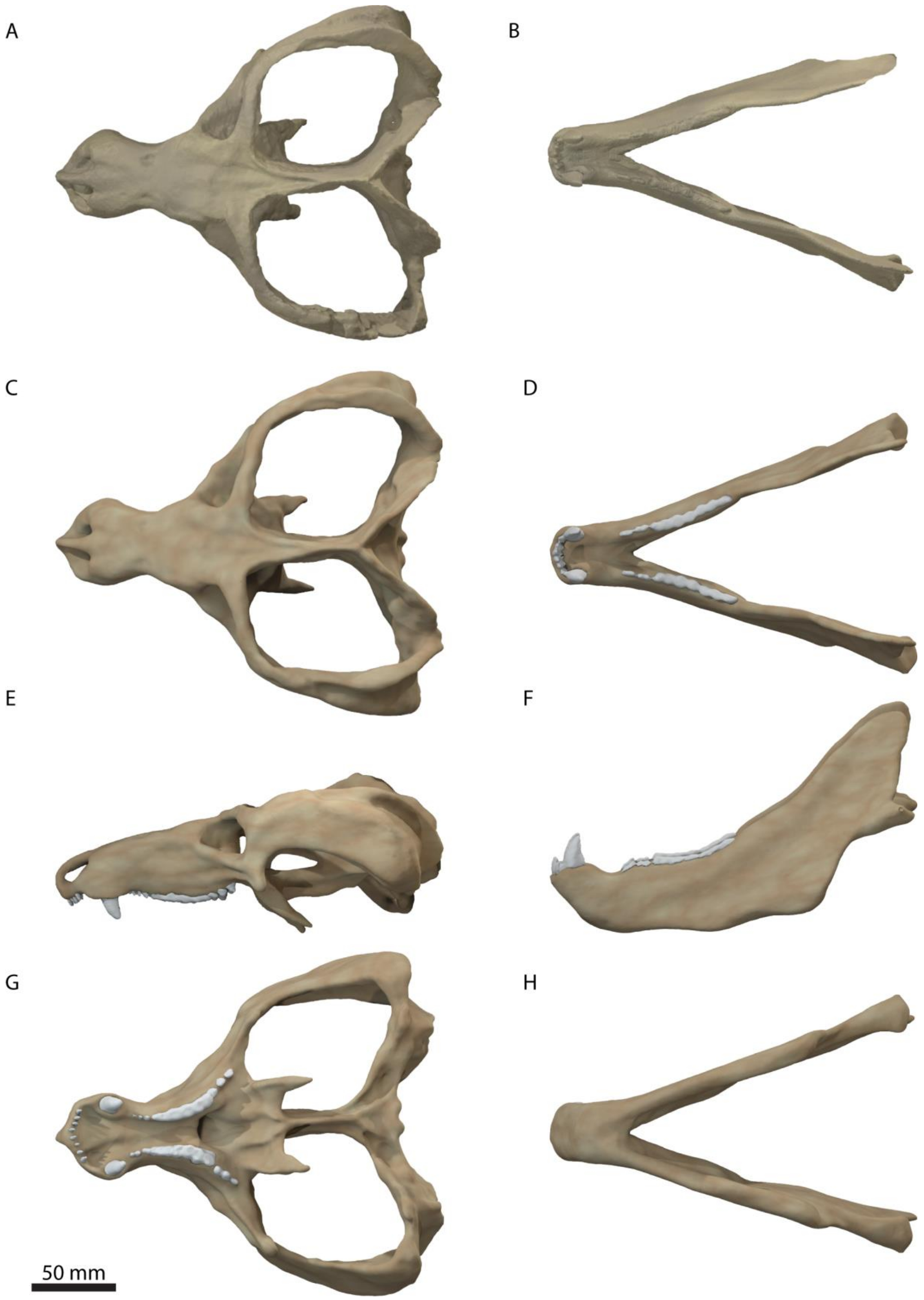

Fig. S2. Restored osteology of *Diademodon tetragonus*. Digital models of the original (A) and restored (C, E, G) skull and the original (B) and restored (D, F, H) lower jaw in (A–D) dorsal, (E, F) left lateral and (G, H) ventral views.
