# Supplementary material for: Morphological evolution of the mammalian jaw adductor complex
Source: Biol Rev Camb Philos Soc. 2016 Nov 23;92(4):1910–40. doi: 10.1111/brv.12314 (PMC6849872; doi:10.1111/brv.12314)
Supplement: Supplementary file 3 — Figure S3. Restored osteology of Probelesodon sanjuanensis. [file BRV-92-1910-s003.pdf]

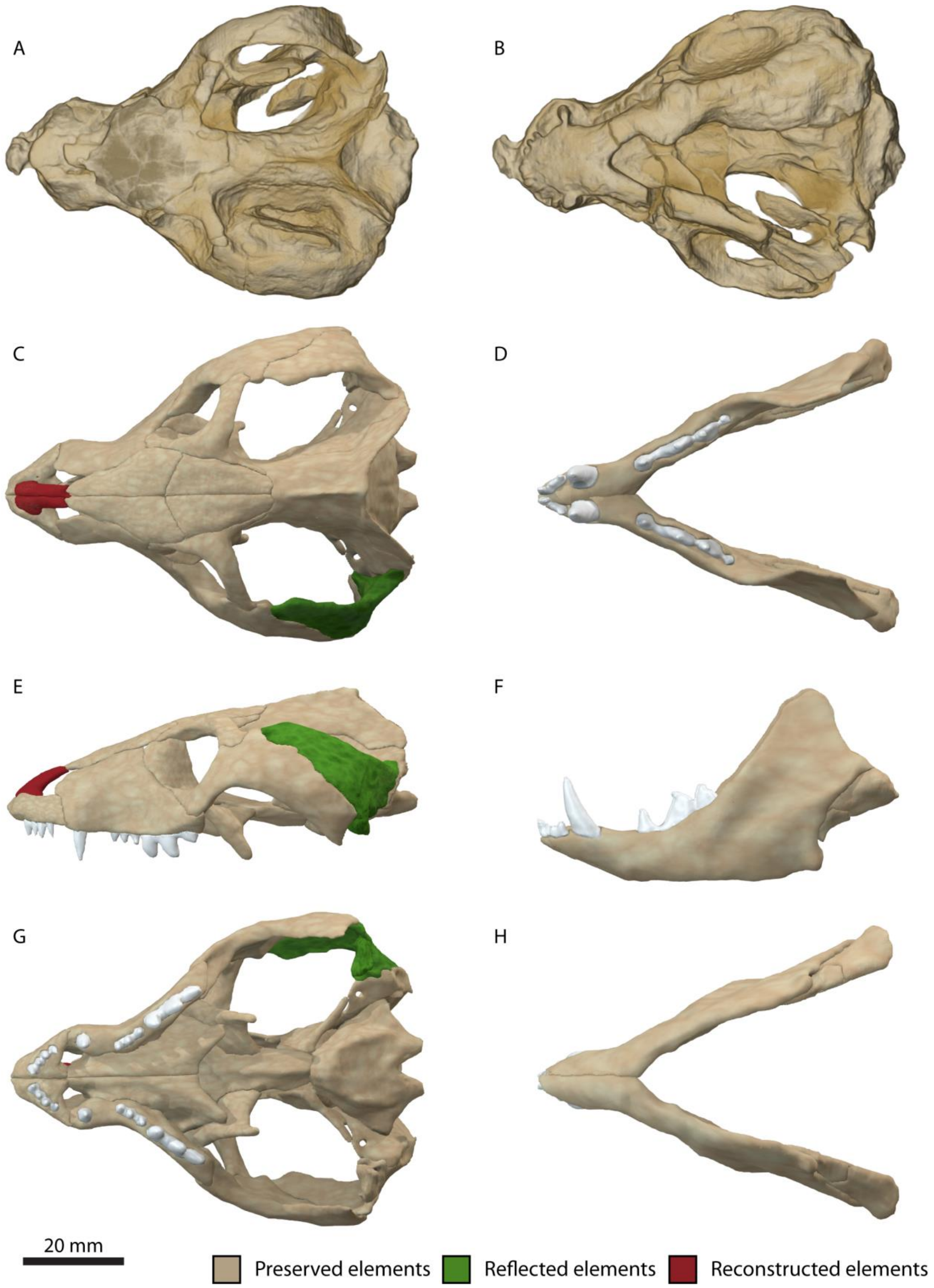

Fig. S3. Restored osteology of *Probelesodon sanjuanensis*. Digital models of the original (A, B) and restored (C, E, G) skull and the restored lower jaw (D, F, H) in (A, C, D) dorsal, (E, F) left lateral and (B, G, H) ventral views.
