# Supplementary material for: Morphological evolution of the mammalian jaw adductor complex
Source: Biol Rev Camb Philos Soc. 2016 Nov 23;92(4):1910–40. doi: 10.1111/brv.12314 (PMC6849872; doi:10.1111/brv.12314)
Supplement: Supplementary file 4 — Figure S4. Restored osteology of Probainognathus sp. [file BRV-92-1910-s004.pdf]

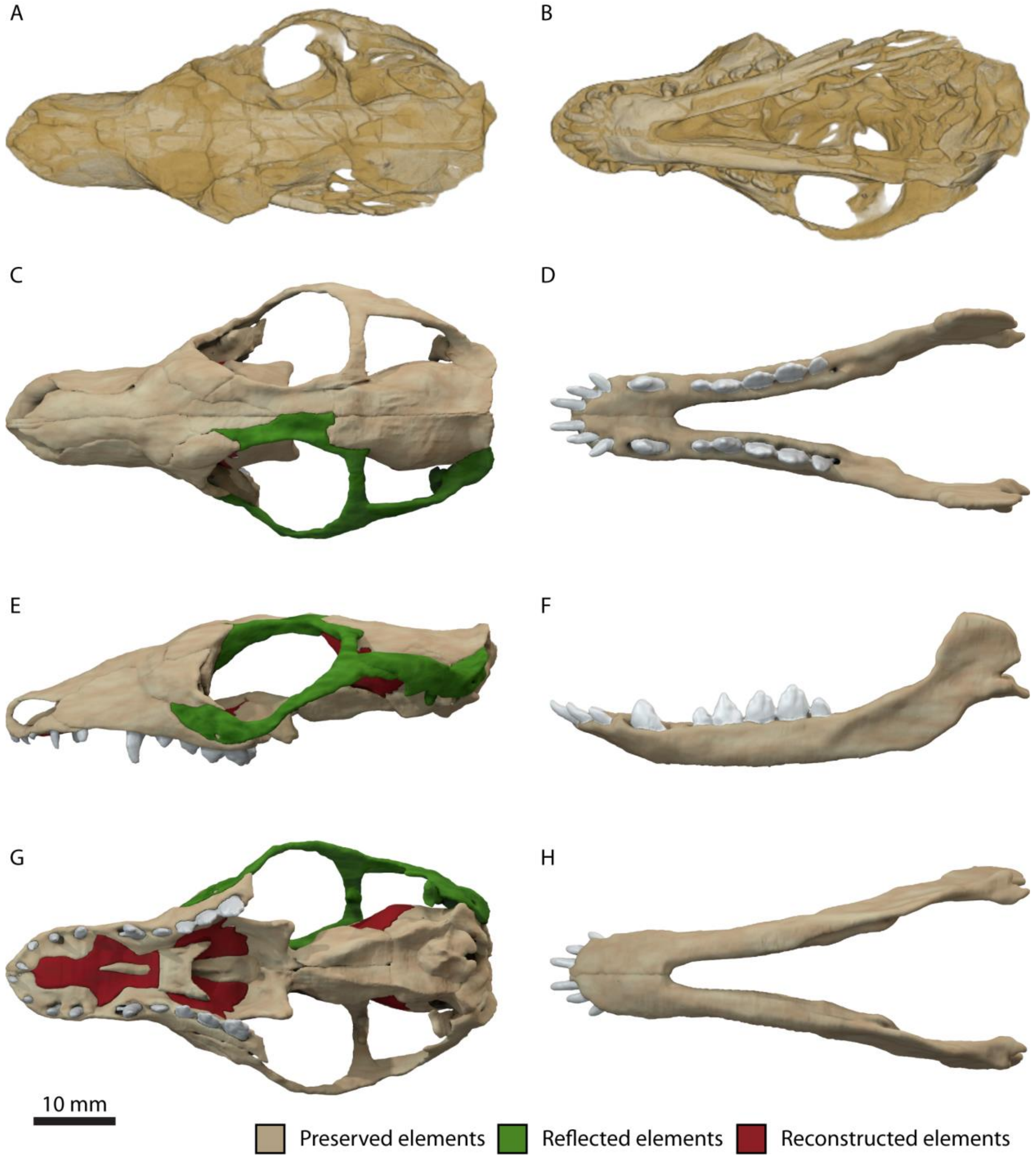

Fig. S4. Restored osteology of *Probainognathus* sp. Digital models of the original (A, B) and restored (C, E, G) and the restored lower jaw (D, F, H) in (A, C, D) dorsal, (E, F) left lateral and (B, G, H) ventral views.
