# Supplementary material for: Morphological evolution of the mammalian jaw adductor complex
Source: Biol Rev Camb Philos Soc. 2016 Nov 23;92(4):1910–40. doi: 10.1111/brv.12314 (PMC6849872; doi:10.1111/brv.12314)
Supplement: Supplementary file 5 — Figure S5. Restored osteology of Morganucodon oehleri. [file BRV-92-1910-s005.pdf]

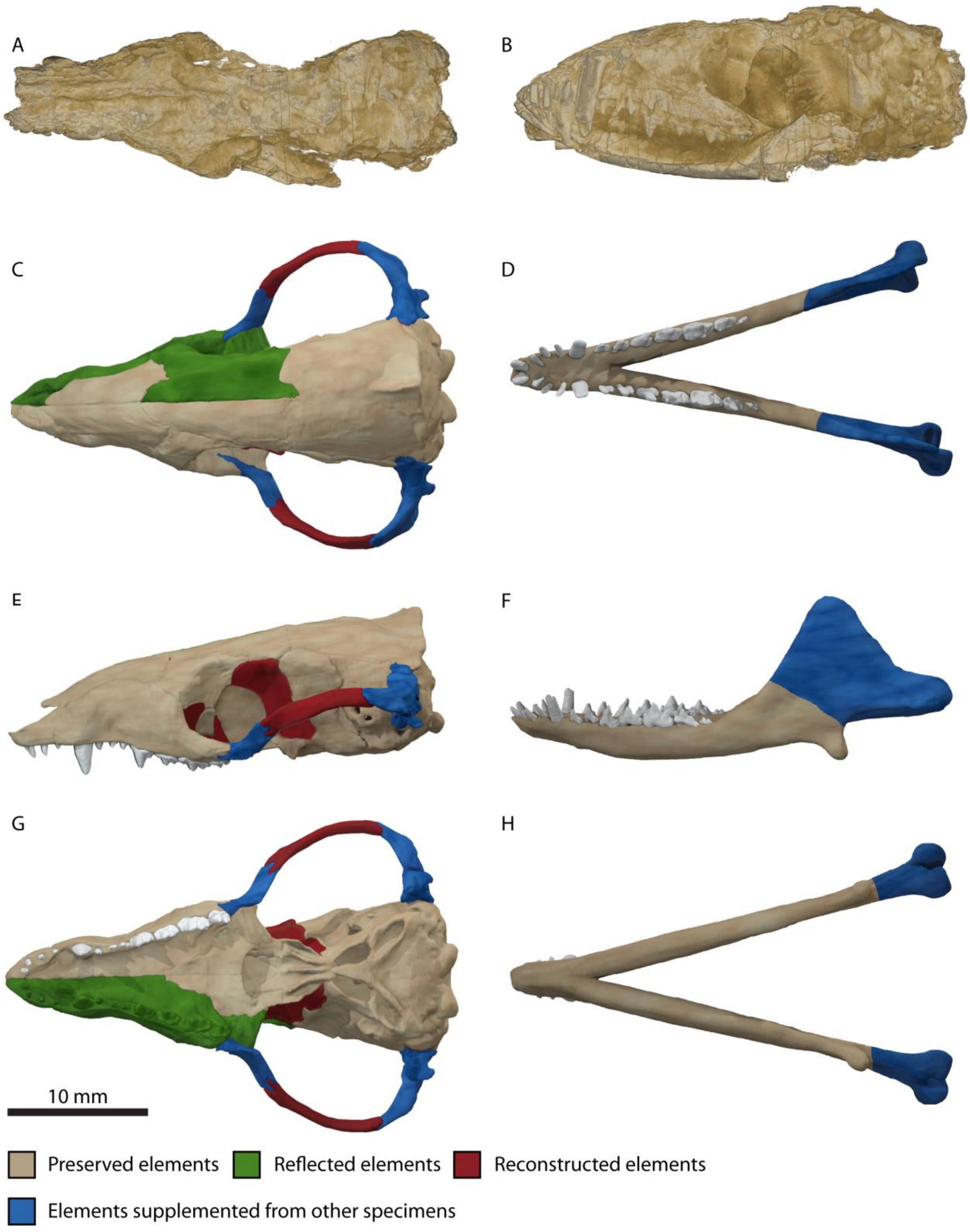

Fig. S5. Restored osteology of *Morganucodon oehleri*. Digital models of the original (A, B) and restored (C, E, G) skull and the restored lower jaw (D, F, H) in (A, C, D) dorsal, (B, E, F) left lateral and (G, H) ventral views. Blue elements indicate regions supplemented from *Morganucodon oehleri* specimen IVPP 8685, or specimens of *M. watsoni* NHMUK PV M 26144, NHMUK PV M 92838 and NHMUK PV M 27410.
