# Supplementary material for: Morphological evolution of the mammalian jaw adductor complex
Source: Biol Rev Camb Philos Soc. 2016 Nov 23;92(4):1910–40. doi: 10.1111/brv.12314 (PMC6849872; doi:10.1111/brv.12314)
Supplement: Supplementary file 6 — Figure S6. Restored osteology of Hadrocodium wui. [file BRV-92-1910-s006.pdf]

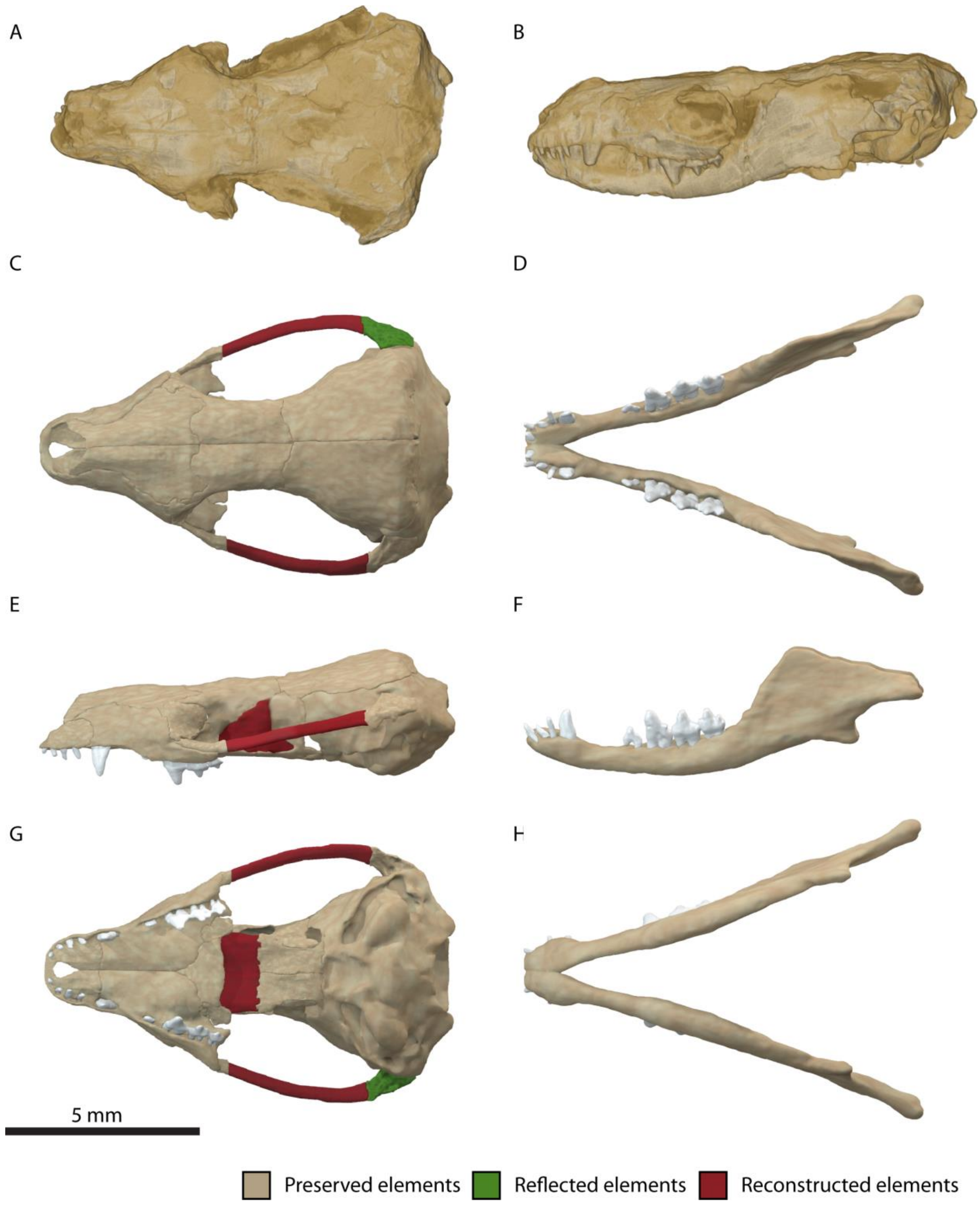

Fig. S6. Restored osteology of *Hadrocodium wui*. Digital models of the original (A, B) and restored (C, E, G) skull and the restored lower jaw (D, F, H) in (A, C, D) dorsal, (B, E, F) left lateral and (G, H) ventral views.
