# Supplementary figures and images for: Morphological evolution of the mammalian jaw adductor complex
Source: Biol Rev Camb Philos Soc. 2016 Nov 23;92(4):1910–40. doi: 10.1111/brv.12314 (PMC6849872; doi:10.1111/brv.12314)

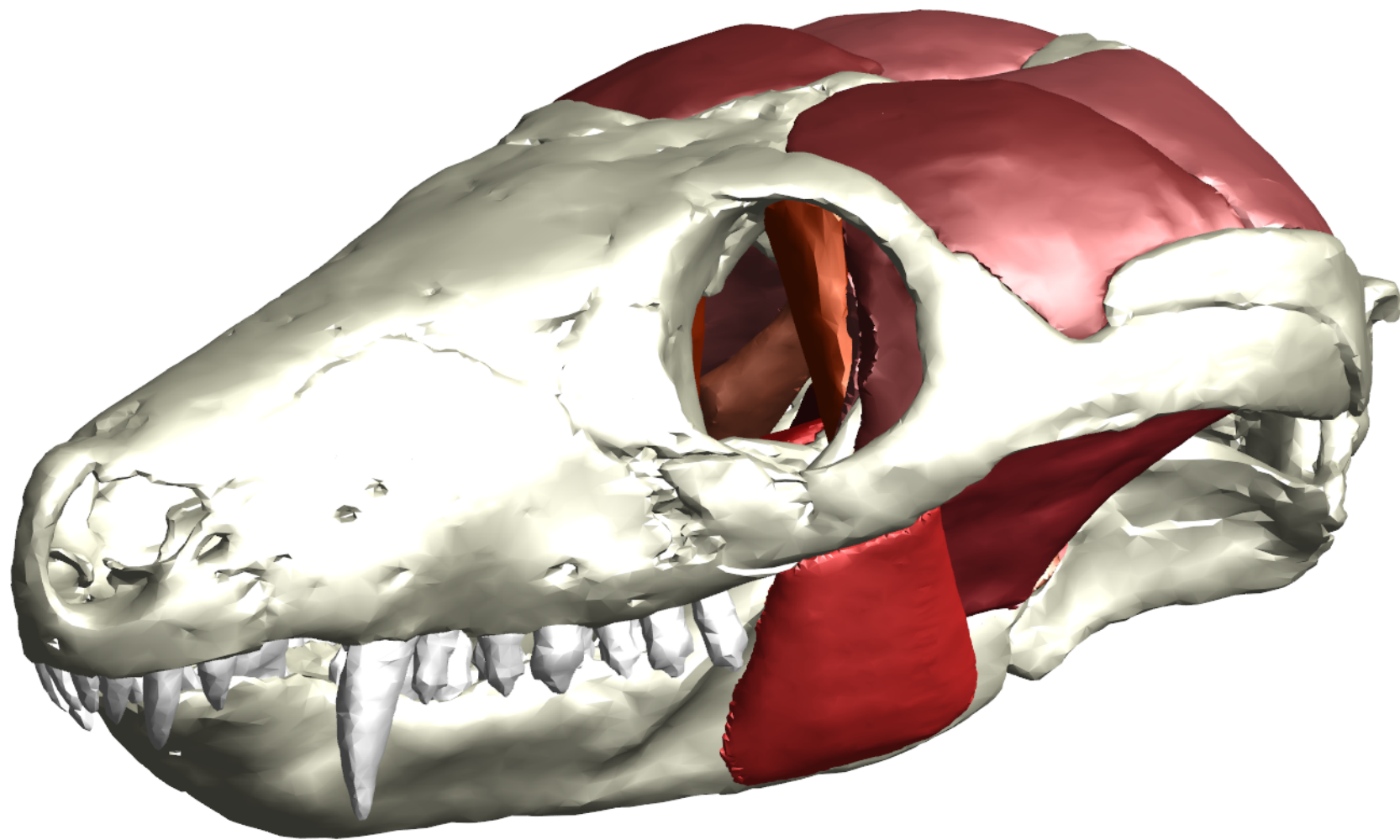

Supplement: Supplementary file 7 — Figure S7. Interactive 3D PDF of Thrinaxodon liorhinus containing the digital model of the restored osteology and the reconstructed musculature. [file BRV-92-1910-s007.pdf]

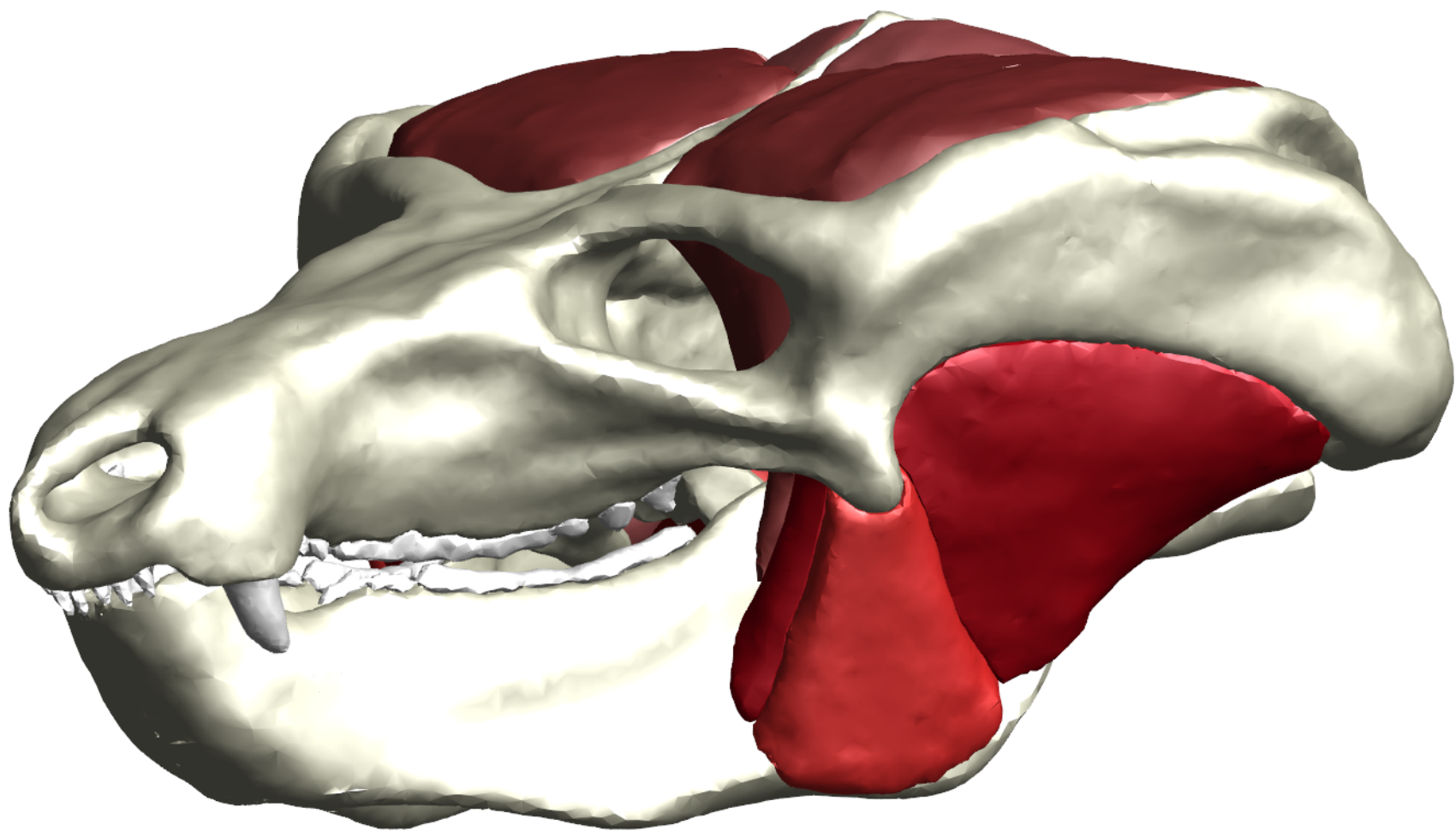

Supplement: Supplementary file 8 — Figure S8. Interactive 3D PDF of Diademodon tetragonus containing the digital model of the restored osteology and the reconstructed musculature. [file BRV-92-1910-s008.pdf]

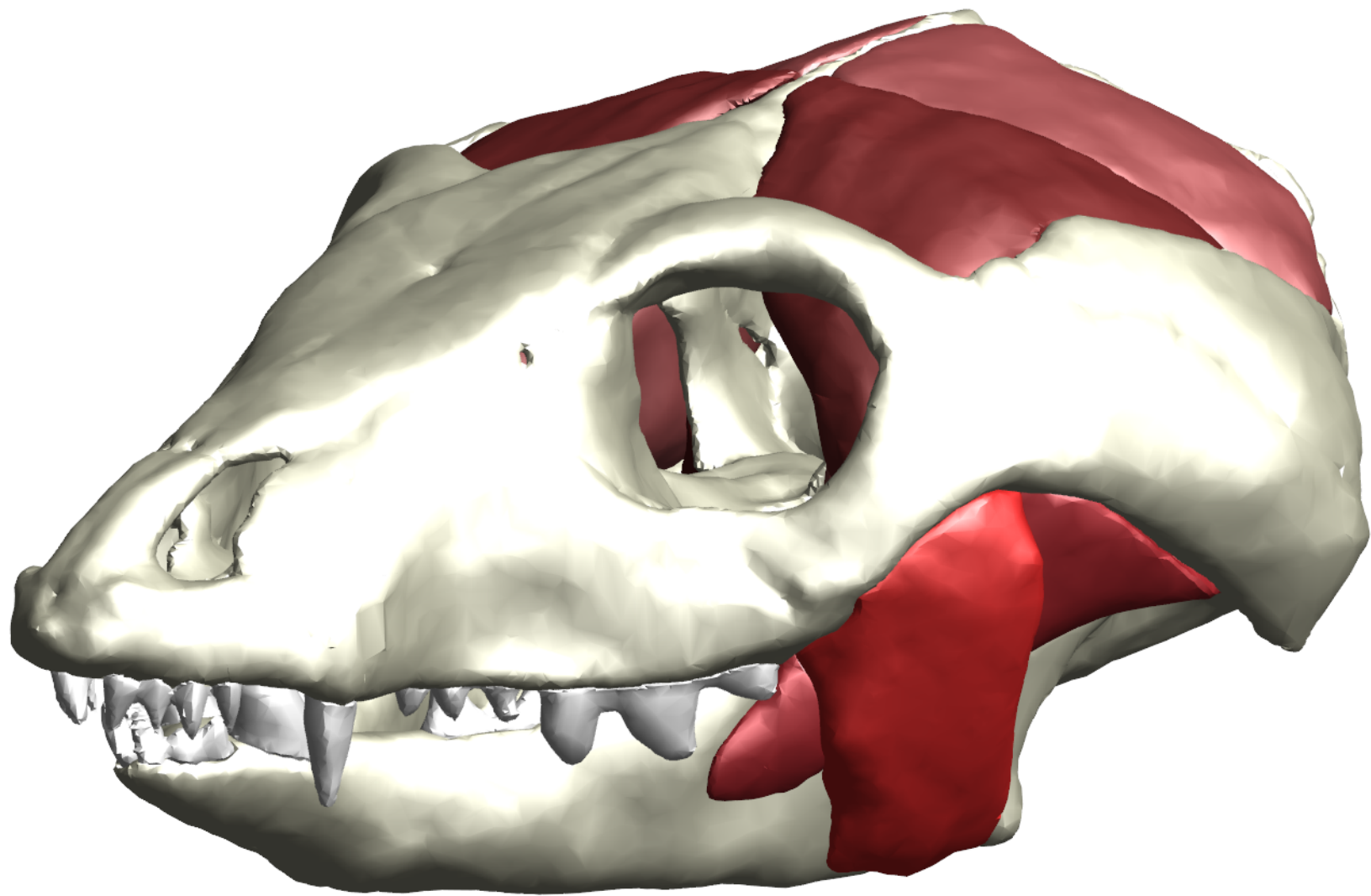

Supplement: Supplementary file 9 — Figure S9. Interactive 3D PDF of Probelesodon sanjuanensis containing the digital model of the restored osteology and the reconstructed musculature. [file BRV-92-1910-s009.pdf]

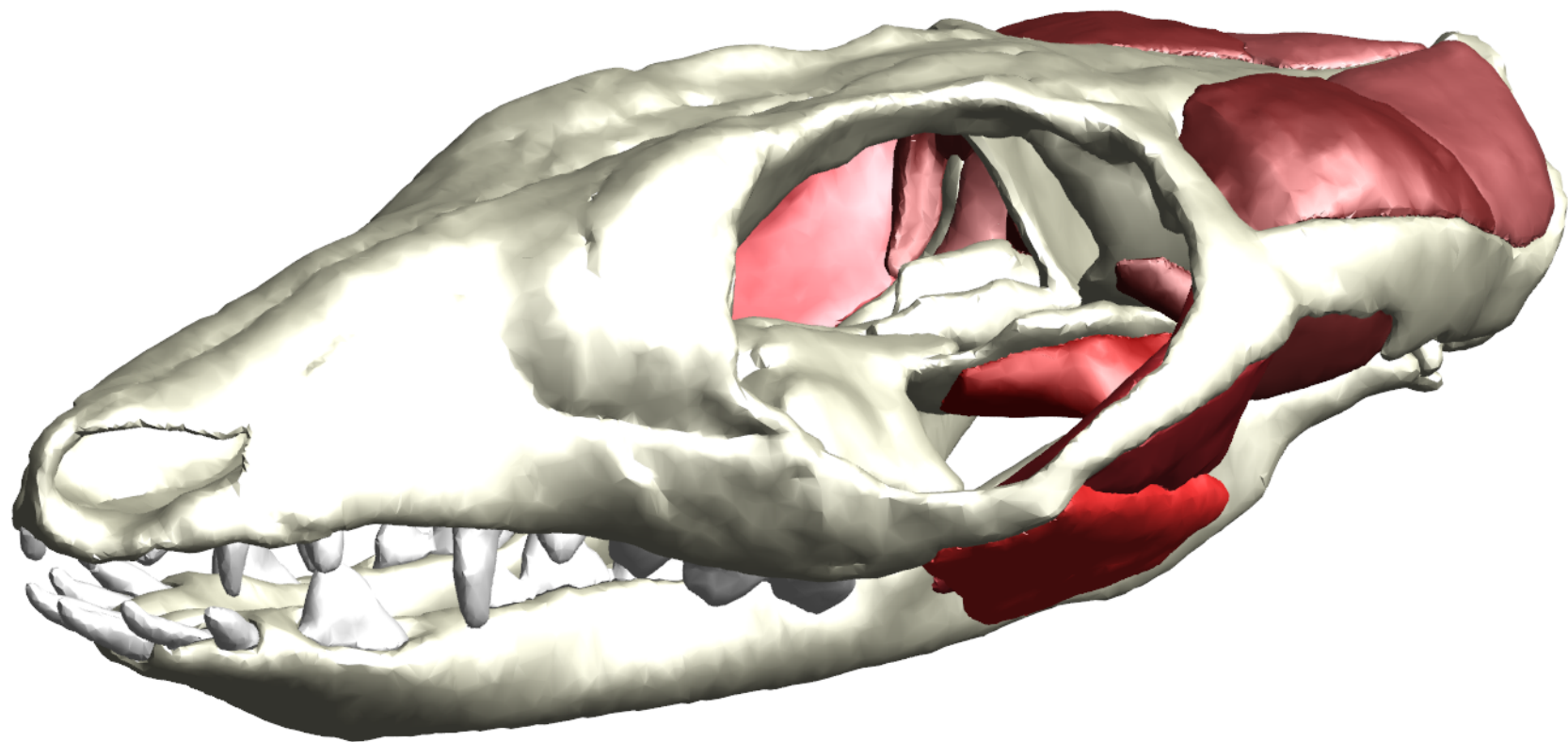

Supplement: Supplementary file 10 — Figure S10. Interactive 3D PDF of Probainognathus sp. containing the digital model of the restored osteology and the reconstructed musculature. [file BRV-92-1910-s010.pdf]

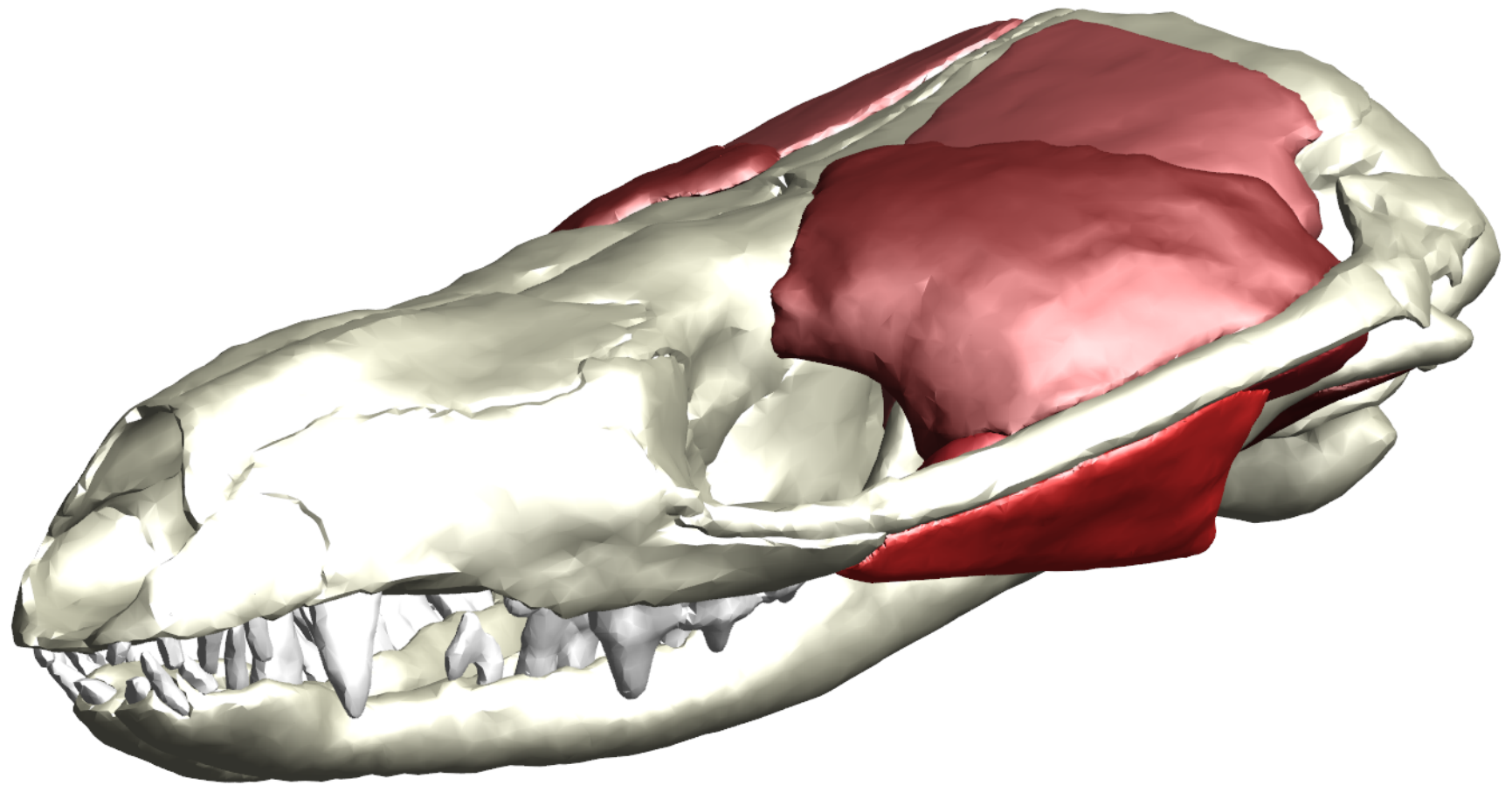

Supplement: Supplementary file 12 — Figure S12. Interactive 3D PDF of Hadrocodium wui containing the digital model of the restored osteology and the reconstructed musculature. [file BRV-92-1910-s012.pdf]

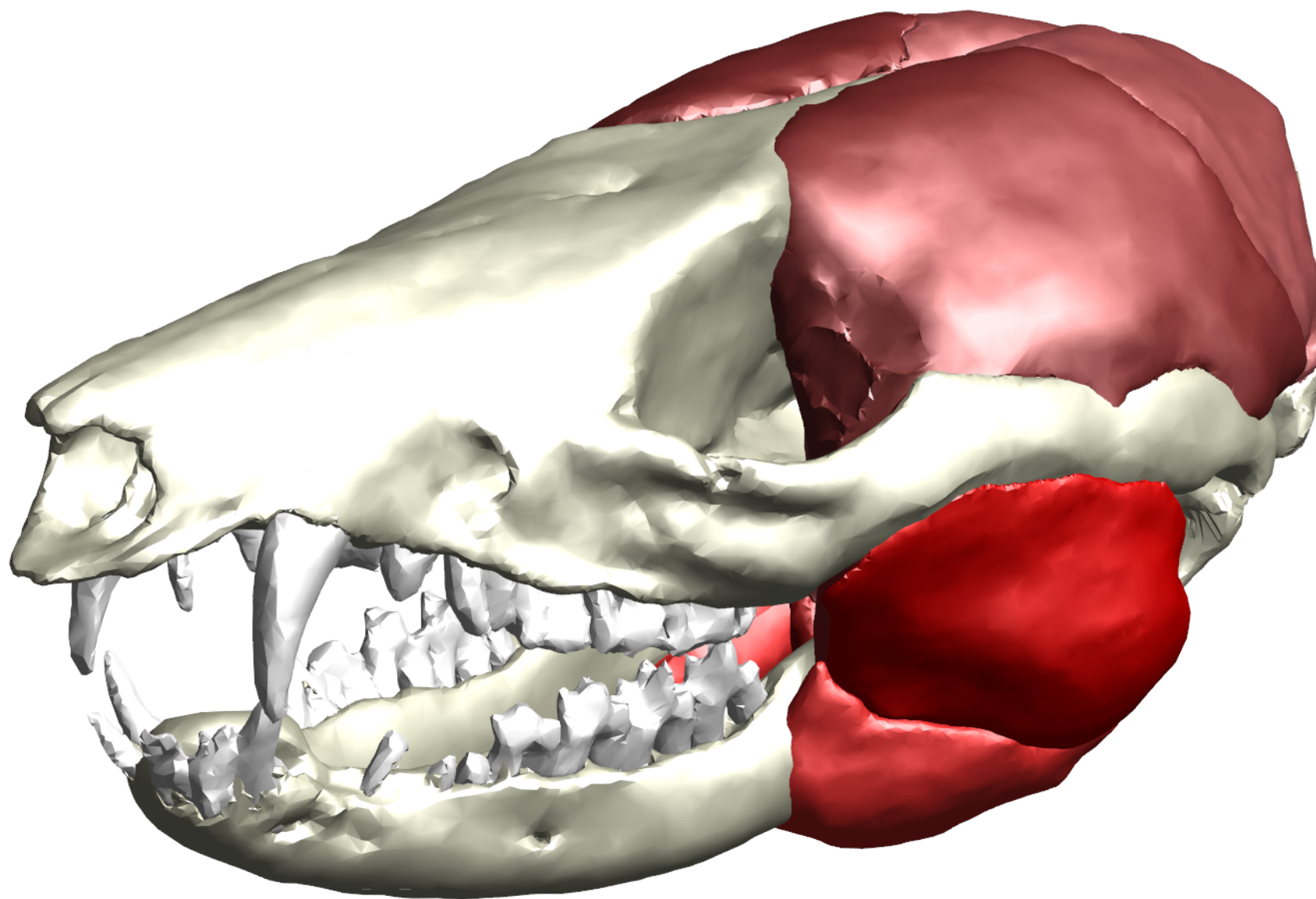

Supplement: Supplementary file 13 — Figure S13. Interactive 3D PDF of Monodelphis domestica containing the digital model of the restored osteology and the reconstructed musculature. [file BRV-92-1910-s013.pdf]
